# Comparative Analysis of Deep Learning Models for Predicting Causative Regulatory Variants

**DOI:** 10.3390/genes16101223

**Published:** 2025-10-15

**Authors:** Gaetano Manzo, Kathryn Borkowski, Ivan Ovcharenko

**Affiliations:** 1 Computational Biology Branch, Division of Intramural Research, National Library of Medicine, National Institutes of Health, 8600 Rockville Pike, Bethesda, MD 20894, USA; gaetano.manzo@nih.gov (G.M.);; 2 Computer Science and Business Management, Case Western Reserve University, 10900 Euclid Ave., Cleveland, OH 44106, USA

**Keywords:** regulatory variants, deep learning models, enhancer activity

## Abstract

Background/Objective: Genome-wide association studies (GWAS) have linked many noncoding variants to complex traits and diseases, but distinguishing as-sociation from causation remains difficult. Deep learning models—particularly CNN- and Transformer-based architectures—are widely used for this task, yet comparisons are hindered by inconsistent benchmarks and evaluation practices. We aimed to establish a standardized assessment of leading models for predicting variant effects in enhancers and for prioritizing putative causal SNPs. Methods: We evaluated state-of-the-art deep learning models under consistent training and evaluation conditions on nine datasets derived from MPRA, raQTL, and eQTL ex-periments. These datasets profile the regulatory impact of 54,859 single-nucleotide polymorphisms (SNPs) across four human cell lines. Performance was compared for two related tasks: predicting the direction and magnitude of regulatory impact in enhancers and identifying likely causal SNPs within linkage disequilibrium (LD) blocks. We addi-tionally assessed the effect of fine-tuning on Transformer-based models and the impact of certainty in experimental results. Results: CNN models such as TREDNet and SEI performed best for predicting the reg-ulatory impact of SNPs in enhancers. Hybrid CNN–Transformer models (e.g., Borzoi) performed best for causal variant prioritization within LD blocks. Fine-tuning benefits Transformers but remains insufficient to close the performance gap. Conclusions: Under a unified benchmark, CNN architectures are most reliable for esti-mating enhancer regulatory effects of SNPs, while hybrid CNN–Transformer models are superior for causal SNP identification within LD. These comparisons help guide model selection for variant-effect prediction in noncoding regions.

## 1. Introduction

Genome-wide association studies (GWASs) have revealed that around 95% of disease-associated genetic variants occur in noncoding regions of the human genome, with causative variants commonly affecting regulatory elements that modulate gene expression [1,2,3]. These regulatory variants can profoundly affect phenotypes and alter disease susceptibility by dysregulating their target genes [4].

Enhancers are regulatory DNA elements that control the timing, location, and intensity of gene transcription. Unlike promoters, which are typically located immediately upstream of transcription start sites (TSSs), enhancers can act both in proximity to the TSS (proximal enhancers) or at distances of tens of kilobases to over a million of basepairs away (distal enhancers), often looping to contact their target promoters [5]. Enhancers are characterized by specific epigenomic signatures, H3K4me1 and H3K27ac histone modifications, lack of H3K4me3 (which marks promoters), and evidence of open chromatin detected by DNase I hypersensitive sites (DHSs) or ATAC-seq [5,6,7,8]. The average length of functional enhancer elements is on the order of a few hundred base pairs, with many clustered in so-called super-enhancers that regulate genes critical for cell identity [9]. Even a single nucleotide polymorphism (SNP) within an enhancer can significantly alter transcription factor (TF) binding affinity, disrupt enhancer–promoter contact, and ultimately impact the level of target gene expression. Many noncoding variants are associated with human diseases and complex traits [10,11]. Recent large-scale studies (e.g., [12]) have demonstrated that enhancer variants are enriched in loci linked to a large number of human diseases including immune-related and neuropsychiatric disorders, underscoring their widespread contribution to disease susceptibility. Importantly, these effects are often cell-type specific, highlighting the necessity of studying enhancers within the appropriate biological context.

Identifying which putative mutations within enhancers are truly functional remains a time-consuming and labor-intensive process. Experimental high-throughput assays such as Massively Parallel Reporter Assays (MPRAs) have accelerated this effort by enabling the simultaneous testing of thousands of candidate enhancer sequences for regulatory activity [13,14]. However, MPRAs remain limited: they are costly, context-dependent, and not yet scalable to systematically test the millions of potential noncoding variants across the human genome. Moreover, as highlighted by recent reviews [15], MPRAs and other perturbation assays need to be interpreted cautiously, as the regulatory activity measured outside of native chromatin environments may not fully reflect endogenous enhancer function.

To overcome these limitations, computational approaches have emerged as complementary strategies to predict enhancer activity and prioritize causal variants. By integrating sequence features, TF binding motifs, chromatin accessibility, and epigenomic annotations, machine learning and deep learning models can predict enhancer activity and assess the potential impact of noncoding variants at scale. Such approaches not only help narrow down candidate variants for experimental validation but also provide insights into the cell-type specificity and regulatory architecture underlying enhancer function, paving the way for systematic interpretation of the noncoding genome [15].

Deep learning has emerged as a transformative approach for predicting the regulatory effects of genetic variants, particularly within enhancer regions. These models harness large-scale genomic and epigenomic datasets to learn complex sequence-to-function relationships, identifying DNA sequence features that influence regulatory activity. Beyond the choice of neural network architecture, the central goal of deep learning in regulatory genomics is to identify recurring patterns, assess their functional relevance, and detect when they are disrupted. The process typically begins with dimensionality reduction of raw input sequences (e.g., DNA), enabling the model to extract key regulatory features such as transcription factor binding sites, promoters, or enhancers. In traditional architectures, such as Convolutional Neural Networks (CNNs), the core principle is to learn hierarchical representations: early layers capture low-level features (e.g., k-mer composition), while deeper layers progressively integrate these into higher-order regulatory signals. In contrast, modern Transformer-based architectures encode features into high-dimensional embeddings and organize them within a latent space that explicitly models dependencies across long genomic distances. Within this latent space, elements that frequently co-occur—such as an enhancer and its cognate promoter—tend to cluster closely, reflecting their functional association. Conversely, a single nucleotide polymorphism (SNP) that disrupts this interaction will increase the latent-space distance between these elements, signaling a loss of regulatory coherence.

Deep learning models can highlight such perturbations either through shifts in embedding proximity or via feature attribution methods, thereby selecting sequence variants with potential regulatory impact.

For instance, CNNs have been successfully applied to detect variants that disrupt TF binding sites or alter chromatin accessibility, offering mechanistic insights into their potential phenotypic consequences. Notable CNN-based models include DeepSEA [16], SEI [17], TREDNet [18], and ChromBPNet [19]. More recently, Transformer-based architectures have demonstrated strong performance in capturing long-range dependencies and modeling cell-type-specific regulatory effects. These models—such as the DNABERT series [20,21], the Nucleotide Transformer family [22,23], Caduceus [24] and Enformer [25]—were pre-trained on large-scale genomic sequences using self-supervised objectives and subsequently fine-tuned for specialized tasks, including predicting DNA methylation patterns, enhancer activity, and the functional impact of disease-associated variants. By integrating contextual information across broader genomic regions, Transformer models offer enhanced resolution for interpreting noncoding variation in a cell-type-aware manner.

Selecting the most suitable model for detecting the regulatory effects of genetic variants remains a significant challenge despite several surveys offering detailed overviews of the deep learning ecosystem in this domain [26,27,28,29]. These surveys have highlighted the unique strengths and limitations of various models. However, they lack a unified framework for assessment. Specifically, existing reviews often fail to benchmark models on a standardized dataset, train or fine-tune them under consistent conditions, and evaluate their performance using uniform criteria. Furthermore, a fundamental difference between benchmarking models on regulatory regions versus regulatory variants is rarely addressed. While regulatory region analyses focus on identifying broader functional elements, regulatory variant assessments require evaluating the impact of specific sequence alterations within these regions, presenting distinct challenges and opportunities for model evaluation. Recent efforts such as DART-Eval [30] have taken necessary steps toward standardizing the evaluation of DNA language models by introducing a comprehensive benchmark that spans multiple regulatory genomics tasks, including variant effect prediction. Their results revealed that state-of-the-art Transformer-based models—including DNABERT-2 and Nucleotide Transformer—often performed poorly at predicting the direction and magnitude of allele-specific effects measured by MPRA. These findings highlight the limitations of current architectures in capturing subtle, functionally meaningful sequence differences introduced by single-nucleotide variants. Motivated by this, we expand the scope of our evaluation to include a broader set of models, incorporating CNN-based approaches such as SEI and TREDNet, which are known for their robustness in modeling local sequence motifs and regulatory element activity.

In this study, we evaluate state-of-the-art deep learning models to predict the effects of genetic variants on enhancer activity in the human genome. Our approach involved curating and integrating nine datasets derived from diverse experimental methodologies, including massively parallel reporter assay (MPRA), reporter assay quantitative trait loci (raQTL), and expression quantitative trait loci (eQTL) studies. These datasets encompass 54,859 single-nucleotide polymorphisms (SNPs) in enhancer regions across four human cell lines.

**Figure 1:**
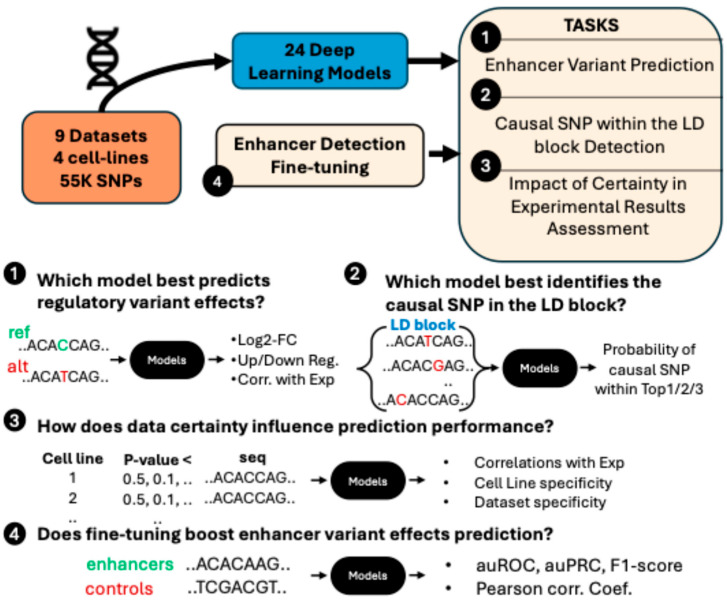
An overview of the evaluation tasks and settings.

We address three major tasks: predicting fold-changes in enhancer activity, classifying SNPs according to their regulatory impact, and identifying causal SNPs within linkage disequilibrium (LD) blocks. We also examine how variability in experimental data influences model performance, underscoring the critical role of data quality. Finally, we demonstrate how enhancer detection models can be repurposed for variant effect prediction, illustrating their utility for interpreting genomic data and prioritizing candidate regulatory variants (Figure 1). To ensure a comprehensive evaluation, we applied several deep learning models to each cell line, systematically exploring a broad spectrum of architectures and hyperparameter configurations.

Our results indicate that CNN models outperform more “advanced” architectures, such as Transformers, on causative regulatory variant detection. However, fine-tuning significantly boosts the performance of Transformer-based architectures, revealing their potential to surpass CNNs under optimized conditions. Moreover, our findings highlight that no single architecture is universally optimal; rather, model selection should be guided by the biological question and the nature of the data being analyzed. For example, convolutional neural networks (CNNs) excel at causative regulatory variant detection, likely due to their ability to capture local motif-level features. By contrast, Transformer-based models benefit substantially from fine-tuning, and under optimized conditions can surpass CNNs, particularly for tasks requiring integration of long-range sequence dependencies.

## 2. Results

### 2.1. Comparison of Enhancer Variant Prediction Models

We evaluated a diverse set of state-of-the-art deep learning models for their ability to predict the effects of genetic variants on enhancer activity in the human genome. These models differ markedly in architecture, parameter count, and the types of data used during training or pre-training. Notably, they were originally developed with distinct objectives in mind. For instance, models like Nucleotide Transformer [22] and DNABERT-2 [21] were designed for broad genomic representation learning, whereas others—such as Geneformer [26] and TREDNet [18]—were tailored for more specific applications.

Geneformer, in particular, is a Transformer-based model initially built to analyze single-cell gene expression profiles. However, it can be fine-tuned for a range of tasks, including chromatin state prediction and in silico perturbation modeling [26]. In our study, we adapted and fine-tuned Geneformer and other models to predict the effects of regulatory variants, leveraging their capacity to capture biological context for accurate assessment of enhancer activity changes (see Section 4, Model Adaptation and Fine-tuning). Importantly, this work compares not only model architectures but also their training foundations—highlighting how differences in pre-training data and objectives can impact downstream performance. To better disentangle these factors, we additionally assessed how the same model performs when applied across different experimental datasets. Despite their original design differences, all selected models were repurposed to predict enhancer variant effects (see Section 4).

Our benchmark spans 54,859 single-nucleotide polymorphisms located in enhancer regions, compiled from nine datasets across four human cell lines: K562, HepG2, NPC, and HeLa. This evaluation provides insights into how both model design and training data influence predictive accuracy for regulatory variant interpretation.

**Figure 2:**
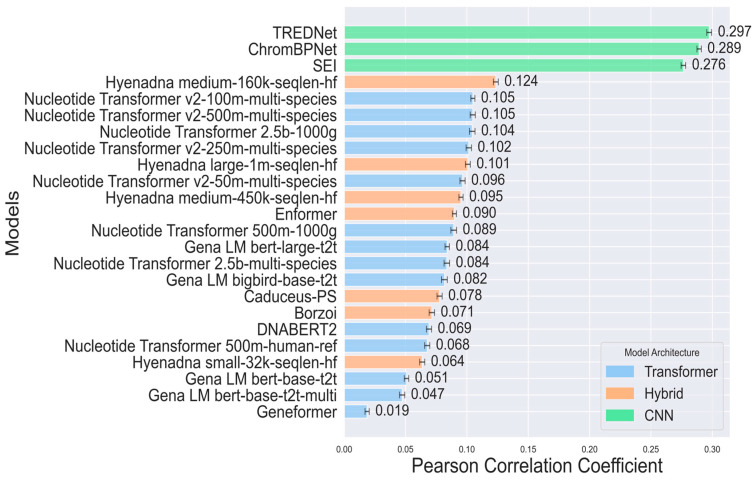
Average Pearson correlation coefficients between model predictions and experimental log2-fold-change for enhancer variant effects across the human genome (9 datasets, 4 cell lines, ~50K SNPs. Bar colors denote model architectures (CNN: green, Transformer: blue, and hybrid: orange). All correlations have *p*-values < 0.05, and error bars show variance.

We performed regression analyses between model predictions and experimental log2-fold-changes for variant effects in enhancers across the human genome sequence. For each dataset, we conducted a separate analysis and averaged the results to provide a robust comparison across experimental settings (Pearson Correlation Figure 2, Spearman Correlation Figure S1, Section 4). We acknowledge that our classification groups together all models that are not purely CNN- or Transformer-based, despite significant architectural differences, particularly in the case of HyenaDNA, which diverges substantially from attention-based models. Nonetheless, our primary objective is to establish a broad guideline for enhancer variant prediction models, rather than to define rigid model categories.

CNN-based models, such as TREDNet, ChromBPNet, and SEI, demonstrate the best alignment with experimentally recorded variant effects, achieving Pearson correlation coefficients of 0.297, 0.289 and 0.276, respectively, while the most accurate Transformer-based model, Nucleotide Transformer v2, scores only at 0.105, well below the level of statistical uncertainty. Note that the reported values represent averages across nine Pearson correlation coefficients (one per dataset). Correlation strength varies by dataset, with some reaching values above 0.8 (Tables S1 and S2).

These findings highlight the effectiveness of CNNs in recognizing spatial and structural genomic patterns essential for predicting enhancer variant effects. Convolutional layers identify genomic features, such as local motifs and epigenetic markers, that influence genetic variant effects [29]. This capability is crucial for capturing the complex genomic relationships underlying gene regulation and enhancer activity. In contrast, Transformer-based models fine-tuned for specific cell lines show lower correlation values (0.019–0.105). Despite their ability to model long-range relationships and complex sequences, they struggle to detect the subtle genomic patterns linked to regulatory variant effects.

This challenge likely stems from the complexity of genomic data, lower granularity in feature detection compared to CNNs, and the extensive data required to fully exploit Transformer capabilities. To bridge the gap between local pattern recognition and long-range dependency modeling, hybrid models combine components such as CNNs, Transformers, and LSTMs. Models like the HyenaDNA series and Borzoi show moderate correlations, with HyenaDNA medium-160k-seqlen-hf achieving the highest among hybrids at 0.124. The integration of diverse components enhances performance compared to Transformer-based models while optimizing computational costs relative to CNN-based models. However, despite their architectural versatility, these hybrid models do not surpass the performance of CNN-based models, highlighting a trade-off between flexibility and focused efficiency (Figure 2 and Figure S1).

**Figure 3:**
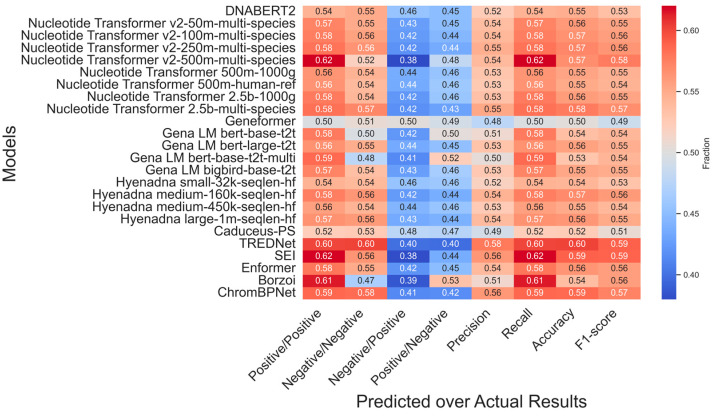
Heatmap of model variant predictions (Predicted) versus experimental values (Actual Results). The color intensity represents the fraction of values predicted as positive (negative) relative to the experimental positive (negative) values. Red indicates higher fractions (desired in the first two columns from the left), while blue indicates lower fractions (desired in the follow two columns). All 9 datasets have been used as input data (~50 SNPs). Precision, Recall, Accuracy and F1-score are indicated in the last four columns.

The classification task provides a deeper understanding of model performance by evaluating true positive and true negative rates in predicting variants that upregulate or downregulate gene expression (Figure 3). Among 54,859 experimental SNPs, approximately half are linked to upregulation and half to downregulation.

CNN-based models, like TREDNet, ChromBPNet, and SEI, excel in predicting variant effects. TREDNet achieves balanced performance with correctly predicted upregulation (0.60) and downregulation (0.60) rates, while SEI leads in upregulation detection (0.62) but struggles with downregulation (0.56). ChromBPNet provides an upregulation of 0.59 and a downregulation of 0.58. Transformer-based models show mixed results. Nucleotide Transformer v2-500m-multi-species matches SEI’s upregulation prediction rate (0.62) but underperforms in downregulation (0.52). DNABERT-2 performs steadily (0.54, 0.55), while Geneformer shows the weakest outcomes. Borzoi achieves a strong upregulation prediction rate (0.61) but falters in downregulation (0.47). Enformer shows moderate, balanced results (0.58, 0.55), while HyenaDNA remains consistent but unremarkable.

**Figure 4:**
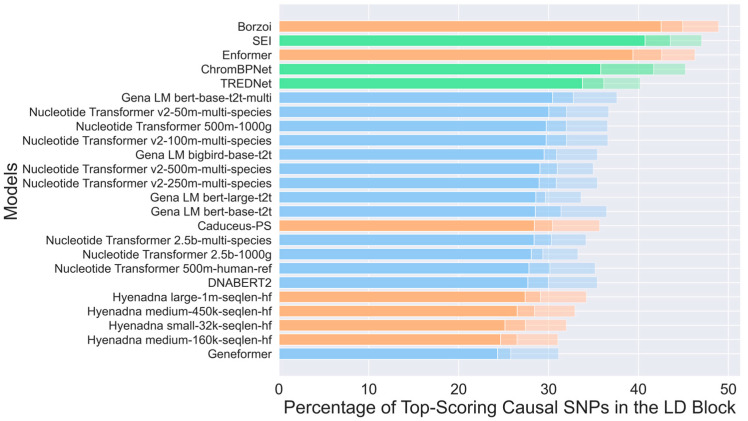
Percentage of top-scoring causal SNPs predicted within the Linkage Disequilibrium (LD) block by various models. Models are grouped by architecture: CNN (green), Transformer (blue), and hybrid (orange). Dataset 2, HepG2 cell line with ~14k raQTL SNPs for input data. Bars are sorted by top-1 performance, with decreasing transparency indicating lower ranks (top 2 and top 3 results are progressively faded compared to top 1).

A crucial aspect of each model is its ability to identify causal SNPs within their respective Linkage Disequilibrium (LD) blocks. This is arguably the most important function of variant classification models, as it enables resolution of causal variants from the large sets of associated SNPs in LD blocks linked to GWAS tag SNPs. To investigate this, we curated a dataset of 14,183 causal SNPs specific to the HepG2 cell line (Figure 4, Dataset 2, see Section 4).

The choice of HepG2 was motivated by three factors: (i) the high quality and comprehensiveness of available enhancer and variant annotations in this cell line; (ii) its extensive use in prior studies, which provides a solid basis for benchmarking and comparison; and (iii) the availability of experimentally supported causal SNPs, which allows us to extract and validate true causative variants for model evaluation. For each causal SNP, we identified associated LD-block SNPs with an r^2^-value above 0.8 within a 500 kbp window, resulting in 263,286 SNPs. This design enables a direct and rigorous assessment of each method’s ability to recover the causal SNP from within an average ~26:1 pool of associated variants.

For each identified SNP, we generated two sequences of 1 kbp: one containing the altered variant and the other containing the reference nucleotide. In both cases, the SNP or reference nucleotides were positioned at the center of the sequence. These sequences were then input into several models to assess their ability to predict causal SNPs by calculating the log2 fold-change between the scores of the alternative sequence and the reference sequence (Section 4). This procedure was repeated for all LD-block associated SNPs and the log2 fold-change score of the known causal SNP was compared to its associated variants. Finally, we computed the percentage of causal SNPs within an LD-block correctly predicted as causal based on their score being either the highest (“top-1” test) or within the top two and three highest scores (“top-2” and “top-3” tests).

The Borzoi model achieved the highest accuracy, correctly identifying 42.5% of causal SNPs, followed by the SEI, Enformer, ChromBPNet and TREDNet models, with 40.7%, 39.4%, 35.2%, and 33.8% in the top1 test, respectively, (Figure 4). The causal variant detection rate increased substantially in the top-2 and top-3 test with Borzoi (44.9%, 48.9), SEI (43.6%, 47.0%), Enformer (42.4%, 46.2%), ChromBPNet (43.4%, 45.1%), and TREDNet (36.1%, 40.1%). Among the Transformer-based models, the Gena LM bert-based-t2t-multi demonstrated the best performance (top-1 30.4%, top-2 32.7%, and top-3 37.5%), marginally surpassing other Nucleotide Transformer and Gena LM variants, which achieved accuracies between 28.0% and 30.0% in top-1 test. The HyenaDNA models, categorized as hybrid architectures, displayed moderate performance, with accuracies ranging from 24.3% to 26.5% in top-1 test, and larger sequence lengths, such as those used in the HyenaDNA large model, led to improved results. The Geneformer model, however, had the lowest accuracy, identifying 24.3% of causal SNPs in top-1 test. Overall, hybrid and CNN architectures outperformed Transformer-based models in this application, underscoring their suitability for tasks involving causal SNP detection.

Overall, models like TREDNet and SEI consistently outperform others in both regression analysis and classification tasks, highlighting the strength of CNN-based architectures in capturing local genomic patterns critical for regulatory variant effect prediction. Hybrid models, such as Borzoi and Enformer, provide balanced performance by combining local pattern recognition with long-range dependency modeling. While they do not surpass CNNs in overall predictive accuracy, they are particularly well-suited for tasks requiring the detection of causal variants within LD-blocks, where modeling long-range context is essential. Transformer-based models, though powerful for learning complex dependencies across extended sequences, currently lack the resolution of CNN and hybrid models for tasks centered on fine-scale motif recognition. However, when optimized, they are best applied to tasks that demand integration of distal regulatory interactions, such as enhancer–promoter contact or tissue-specific regulatory architecture. Based on these observations, we suggest a two-step strategy: first, apply CNN-based models to efficiently identify high-risk variants across the genome, and then leverage hybrid models such as Borzoi to resolve the causal SNPs within the corresponding LD blocks, where long-range context plays a decisive role.

### 2.2. Impact of Certainty in Experimental Results

The results of experimental assays of regulatory variants depend on the selected degree of certainty in separating significant and insignificant variant effects. To investigate the impact of experimental data significance on correlation with modeling results, we analyzed the top models for each architecture type: TREDNet (CNN), HyenaDNA (hybrid), and Nucleotide Transformer (Transformer). The experimental data was binned using the following significance thresholds, *p* < 0.5, *p* < 0.1, *p* < 5 × 10^−5^, *p* < 10^−5^.

**Figure 5:**
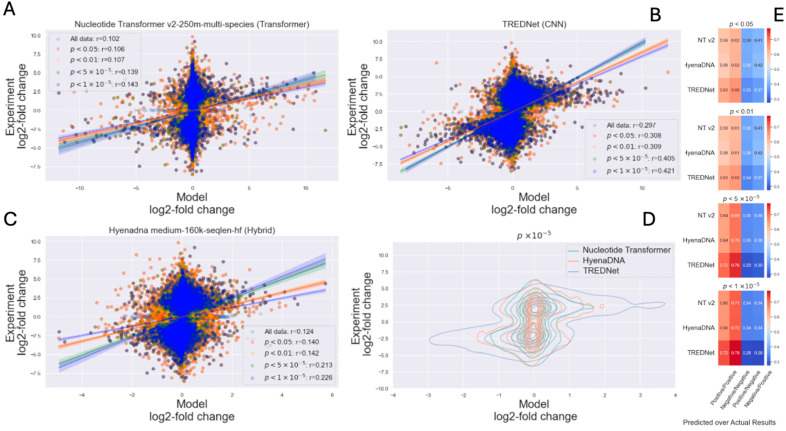
Model performance relative to experimental data significance, using top models from each architecture category. (**A**–**C**) show scatter plots for TREDNet, HyenaDNA, and Nucleotide Transformer, with the correlation between model predictions and experimental results as significance improves. (**D**) compares model predictions for highly significant variants. (**E**) presents heatmaps of classification metrics at different *p*-value thresholds, highlighting performance variations in identifying positive/negative outcomes across architectures.

The results demonstrate that models align better with experimental findings when applied to variants with higher statistical significance. For instance, data points with greater experimental confidence show increased clustering in the first and third quadrants, representing cases where both experimental and predicted results are either positive (upregulation) or negative (downregulation) (Figure 5A–C and Figures S2 and S3a–d).

Among the evaluated architectures, HyenaDNA exhibited the greatest improvement in predictive accuracy with the increase in the statistical significance of experimental results (from all data 0.124 to 0.226 with *p* < 10^−5^, Figure 5C). However, TREDNet consistently displayed superior overall performance, with a higher density of points aligning with experimental outcomes (from all data 0.298 to 0.421 with *p* < 10^−5^, Figure 5B). This trend is further illustrated in the density plots, where TREDNet exhibits increased clustering in the first and third quadrants, demonstrating stronger correlations between model predictions and experimental values compared to both hybrid and Transformer models (Figure 5D). Moreover, the correlation coefficients across varying significance thresholds highlight TREDNet’s robustness, achieving the highest values under all conditions. True positives and true negatives increase with the significance of the experimental results (positive/positive 0.63, negative/negative 0.66, with *p* < 0.05; positive/positive 0.72, negative/negative 0.78, with *p* < 10^−5^), with the hybrid model and the Transformer model following in performance (Figure 5E). The rest of models’ performance relative to experimental data significance can be found in Figures S2 and S3a–d. The correlation between predictions and experimental data improves notably as values around zero are removed, confirming that high-uncertainty regions reduce the interpretability and strength of correlation (Figure S4).

Given the strong cell-line specificity of enhancers [31], determining whether model performance depends on the type of cell line used is essential for understanding their predictive capabilities and limitations. For this, we evaluated whether a model fine-tuned for a specific cell line demonstrates superior performance compared to models from other architectures, such as CNNs, which often rely on generalization rather than cell-line-specific adaptation. Indeed, Transformer-based models, pre-trained on extensive datasets and fine-tuned for targeted tasks, can offer both flexibility and accuracy, making them well-suited for capturing the unique regulatory landscape of cell lines. In contrast, CNN-based models, such as TREDNet and SEI, though highly effective for specific tasks, could require additional steps, such as retraining or the addition of specialized layers, to adapt their outputs for cell-line-specific predictions. However, the results indicate that TREDNet and SEI remain the most effective models for predicting variant effects across different cell lines, even after model fine-tuning (Pearson Correlation Table 1, Spearman Correlation Table S1, Dataset-wise Sup Table S2 and Sup Table S3). For example, the Nucleotide Transformer v2-250m-multi-species model aligns with experimental results in K562 and HepG2 (0.1998 and 0.11) but performs less effectively in NPC and HeLa cells. This variability could stem from differences in pre-training datasets—such as multi-species versus human-specific data—and the fine-tuning approach.

Notably, the hybrid model Enformer, which integrates features from Transformers and CNNs, performs particularly well in HeLa cells (0.245), ranking second only to SEI (0.279). As observed in the previous test, CNN-based models demonstrate superior performance, showcasing robustness across cell-line variants. We also assess the impact of the certainty of experimental results, SEI, which does not require training or fine-tuning for specific cell lines, outperforms fine-tuned models. TREDNet, likely benefiting from its second-phase training tailored to the specific cell line, achieves the best overall performance, further highlighting its ability to leverage cell-line-specific information effectively.

### 2.3. Enhancer Detection Models for Variant Effect Assessment

Models designed to predict variant effects rely primarily on the change in confidence when detecting the enhancer region after introducing the variant.

Next, we investigated whether improving the accuracy of enhancer sequence detection enhances predictions of variant effects. While SEI, Borzoi, and Enformer do not require fine-tuning, TREDNet incorporates a two-phase training despite being a CNN-based model. The second phase can be considered fine-tuning [18].

**Figure 6:**
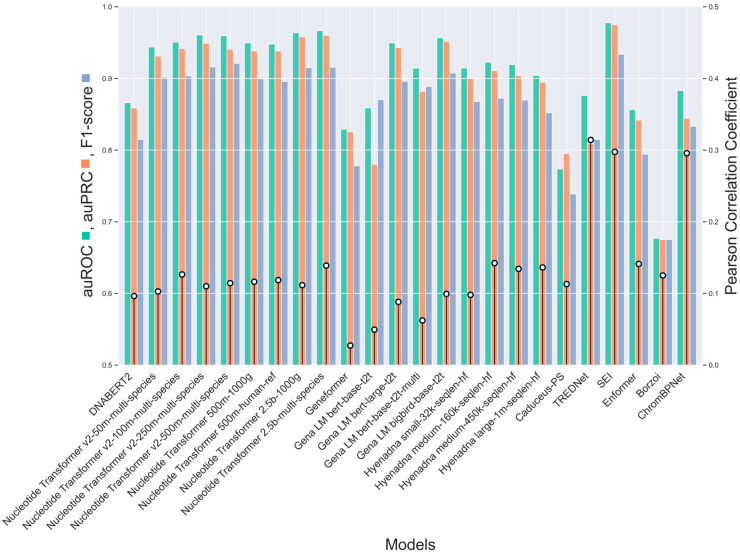
Performance comparison of different models for enhancer detection in HepG2 cell across multiple metrics. Left axis: Area Under the Receiver Operating Characteristic Curve (auROC, green bars), Area Under the Precision-Recall Curve (auPRC, orange bars), and F1-score (blue bars). Right axis: Pearson Correlation Coefficient (black pins).

Transformer-based architectures, particularly Nucleotide Transformer models pre-trained on multi-species datasets, emerge as strong performers in enhancer classification (Figure 6). Models such as Nucleotide Transformer v2-250m-multi-species and Nucleotide Transformer 2.5b-multi-species consistently demonstrate high accuracy, as indicated by their auROC and auPRC metrics (auROC: 0.967, 0.963; auPRC: 0.958, 0.951). However, the correlation between the models’ outputs and the MPRA experimental results, which measure the fold-change effect between reference and alternative sequences, remains relatively low (ranging from 0.112 to 0.15). SEI stands out as the top performer in detecting enhancer activity, achieving the highest auROC (0.974) and auPRC (0.971) scores among all models. It is also the second best for the correlation between model outputs and experimental results (0.295). TREDNet demonstrates strong performance with a higher correlation (0.318), while balancing high auROC (0.872) and auPRC (0.814) scores. The HyenaDNA models show competitive classification metrics, with the Hyenadna medium-160k-seqlen-hf and Hyenadna large-1m-seqlen-hf versions attaining notable auROC (0.933, 0.904) and auPRC (0.921, 0.893) scores. However, their Pearson correlation coefficients are moderate (0.152, 0.151), indicating that while these models classify enhancer activity effectively, further optimization is needed to improve their predictive accuracy for variant-specific effects. Borzoi performs the weakest in detecting enhancer regions (auROC: 0.672, auPRC: 0.65), while Geneformer fares poorly in detecting variants (auROC: 0.027). Yet, this weak enhancer detection performance doesn’t translate in inaccurate detection of causal variants, as Borzoi outperforms all other methods in this critical test (Figure 6).

**Figure 7:**
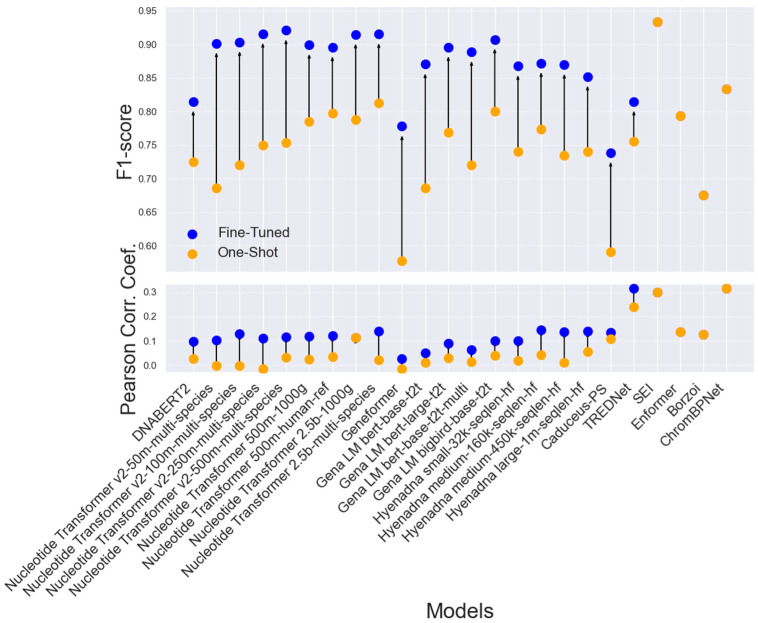
Comparison of F1 score (**top**) and Pearson Correlation Coefficient (**bottom**) between fine-tuned (blue dots) and one-shot (orange dots) models across various architectures, evaluated on Dataset 2, HepG2 cell line, (14,183 SNPs). Arrows indicate the changes in performance between fine-tuned and one-shot implementations for each model.

Fine-tuned models consistently outperform their one-shot counterparts, achieving F1 scores between 0.85 and 0.92, compared to 0.70 to 0.80 for one-shot models (Figure 7, top). This improvement in performance is reflected in the correlation between model predictions and experimental results, particularly in detecting enhancer variants (Figure 7, bottom). The performance enhancement is consistent across most architectures, with CNN-based models showing remarkable results even without fine-tuning. However, SEI, which is available only in its ‘one-shot’ implementation, achieves a high F1 score and correlation, surpassing many fine-tuned Transformer models also when the models were trained on the difference cell-line than the tested dataset (Figure S6). Additionally, some Transformer-based models, such as Nucleotide Transformer 2.5 b–1000 g, show no improvement in detecting enhancer variants.

## 3. Discussion

Our evaluation of deep learning models highlights the strengths and limitations of different architectures in predicting the effects of genetic variants on enhancer activity. CNN-based models, such as TREDNet, ChromBPNet, and SEI, outperform Transformer-based and hybrid architectures in both regression and classification tasks. Notably, Borzoi, a hybrid model, excels in causal SNP prediction, leveraging both local and global genomic features critical for regulatory variant effect prediction.

### 3.1. CNNs-, Transformers-, and Hybrid-Based Models

TREDNet and SEI achieve superior performance in regression (Figure 2) and classification tasks (Figure 3). TREDNet and ChromBPNeT demonstrate balanced predictive capability for both upregulated and downregulated variants, while SEI excels in detecting upregulated variants. CNNs effectively capture local genomic patterns like motifs and epigenetic markers, making them well-suited for enhancer activity analysis. Their simpler architecture yields high Pearson correlation coefficients (0.297 for TREDNet, 0.289 for ChromBPNet, and 0.276 for SEI). However, their inability to model long-range dependencies limits performance in tasks requiring broader sequence context.

Transformers, including the Nucleotide Transformer and DNABERT-2, capture long-range dependencies but struggle with fine-grained regulatory signals. They underperform in enhancer variant effect prediction, with accuracies between 28.0% and 30.0% in causal SNP identification (Figure 4). These models require substantial training data, and their performance could improve with expanded datasets or refined focus on regulatory signals.

Hybrid models, such as HyenaDNA and Borzoi, integrate CNNs, Transformers, and LSTMs to balance local and long-range dependencies. While HyenaDNA shows moderate correlation with experimental results, Borzoi outperforms other models in causal SNP detection (Figure 4), making it valuable for GWAS analysis. However, hybrid models do not consistently surpass CNNs in overall predictive accuracy and should be assessed based on task-specific requirements.

### 3.2. Impact of Experimental Data Quality and Model Generalization

The reliability of model predictions is closely tied to the quality of experimental data. Model alignment with experimental results improves as statistical confidence in variant effects increases (Figure 5 and Figure 6). TREDNet, for instance, maintains high predictive accuracy even under varying significance thresholds, emphasizing the robustness of CNN-based models. When working with high-quality data—for example, datasets with clean sequencing reads, low background noise, strong signal-to-noise ratios, and highly significant peaks or variants (low *p*-values, e.g., <0.001)—CNN models are the preferred choice, as they excel at capturing local regulatory patterns with high accuracy. In contrast, when datasets consist of a mixture of quality levels—such as variable read depth, moderate noise, less stringent peak calls, or broader distributions of statistical confidence—hybrid models like HyenaDNA are recommended, as they maintain robust performance under more heterogeneous conditions. Finally, in cases where very large datasets are available (e.g., >100,000 sequences), fine-tuning Transformer-based models such as Nucleotide Transformer v2 becomes advantageous, as these architectures are able to exploit scale to learn complex dependencies and integrate long-range regulatory interactions more effectively. These findings suggest that enhancing experimental rigor and statistical refinement can reduce uncertainty in variant effect assessments, leading to more reliable predictions.

In addition to data quality, model generalization across different cell lines is a critical factor. Transformer models fine-tuned for specific cell lines can capture regulatory landscapes but often underperform compared to CNNs. For instance, the Nucleotide Transformer v2-250m-multi-species achieves reasonable results in K562 and HepG2 but struggles in NPC and HeLa, likely due to pre-training limitations. In contrast, CNN models generalize well across cell lines without requiring fine-tuning, further demonstrating their robustness in variant effect prediction. Enhancer detection models, such as the Nucleotide Transformer v2-250m-multi-species, achieve high classification accuracy (auROC: 0.967, auPRC: 0.958), yet their predictions show weak correlation with experimental results (Figure 6). CNN-based models, particularly SEI, perform well in both enhancer detection and variant effect prediction. While fine-tuning improves Transformer performance, SEI, despite its one-shot implementation, still outperforms many fine-tuned Transformers (Figure 7).

A notable advantage of Transformer architectures lies in their potential for zero-shot applications (Figure 7), where models can be applied directly without additional training or fine-tuning. In real-case scenarios, this property makes Transformers highly attractive for tasks where training data are scarce or unavailable. At present, the zero-shot models tested here do not yet match the performance of CNN-based approaches, but we consider this a temporary limitation. As Transformer models continue to grow in size and scope—incorporating more comprehensive pre-training across diverse cell types, tissues, and species—they are expected to increasingly capture the regulatory information needed for accurate variant effect prediction in zero-shot settings.

In conclusion, our findings suggest that SEI and TREDNet are the most effective models for predicting the effects of genetic variants on enhancer activity. Hybrid models like Borzoi demonstrate strong potential in extracting causal SNPs from GWAS data, showcasing their value in applications that require the integration of both local and global sequence contexts. Transformer-based models, on the other hand, are particularly effective at identifying enhancer regions due to their ability to capture long-range dependencies, although they still face challenges with fine-grained genomic features and often require substantial data for optimal performance. These results highlight the strengths of different model architectures for various aspects of regulatory variant prediction and underscore the need for further optimization to enhance their performance. Future work should aim to improve feature detection in Transformer models and refine the flexibility of hybrid architectures to better capture both local and long-range genomic dependencies.

## 4. Materials and Methods

### 4.1. Data Pre-Processing

The datasets from HepG2, K562, NPC, and HeLa cell lines underwent a standardized preprocessing pipeline to ensure consistency and feature extraction for downstream analysis. Two main pipelines were applied: one for training and testing enhancer classifier models, such as Nucleotide Transformers and DNABERT-2, and another for mutagenesis experiments. For the enhancer classification pipeline, 1kb sequences were extracted for both enhancer (positive) and control regions. To maintain consistency across models, all nucleotides were capitalized, as some models are case-sensitive. Unknown nucleotides marked as “N” were removed from the sequences. The datasets were then split into training, validation, and test sets, and tokenized using the corresponding model’s tokenizer with a maximum length of 512 tokens. Labels were generated to distinguish between positive and control sequences. In the mutagenesis experiment pipeline, the reference and alternative nucleotide locations were extracted for each row of the dataset. For each nucleotide of interest, a 1kb sequence was generated with the target nucleotide positioned in the center. Chromosome and position information, along with reference and alternative alleles, were also extracted. While longer input sequences generally lead to improved correlation with experimental measurements—indicating that additional genomic context enhances predictive power—the signal-to-noise ratio tends to decrease at higher lengths, suggesting that the additional input may also introduce irrelevant or noisy features that affect model robustness (Figure S5). Log2 activity metrics, such as fold-change values or effect sizes, were calculated to assess regulatory activity. Additionally, signed *p*-values were derived from raw or adjusted *p*-values to capture both the statistical significance and directionality of the regulatory effects.

The preprocessing step ensured that variations in dataset structure did not interfere with downstream analysis. To further enhance consistency, hg19 genomic coordinates were used for variant mapping, and enhancer activity metrics were uniformly applied across datasets. Differences in expression or activity levels between experimental conditions, such as mutant versus control or modern versus archaic sequences, were computed when applicable. Ultimately, all datasets were converted into a standardized format that included chromosome information, positions, reference and alternative alleles, log2 activity ratios, signed *p*-values, raw *p*-values, and other relevant regulatory features.

### 4.2. Model Adaptation and Fine-Tuning

The objective of fine-tuning is to adapt models originally designed as enhancer classifiers to assess the impact of SNPs on enhancer activity. By introducing variant sequences, we evaluate how nucleotide changes affect model scores. To achieve this, we fine-tuned pre-trained DNA language models—including Geneformer (initially developed for scRNA-seq data)—as enhancer classifiers (Table 2).

Sequences were preprocessed as described earlier, with a length of 1 kbp, balancing genomic context and model compatibility. This length also reflects the average enhancer size of ~400 bp. When adapting models like Enformer (196 kb input) and Borzoi (512 kb input), which do not accept arbitrary sequence lengths, we retained their full input size. The 1 kb variant-centered sequence was inserted into representative genomic contexts of the appropriate size, and predictions were averaged over multiple such contexts. Yet, for evaluation settings like Borzoi’s sliding window analysis where the method natively supports shorter sequences, we used 1 kb inputs directly.

Most models were loaded from the Hugging Face Hub using AutoTokenizer and AutoModelForSequenceClassification. Exceptions such as TREDNet, Enformer, SEI, and Borzoi were sourced from GitHub and required custom pipelines for preprocessing and inference.

Fine-tuning was performed using the Hugging Face Transformers library for flexible training and evaluation. Models such as HyenaDNA, Geneformer, Gena-LM, DNABERT-2, and Nucleotide Transformers were trained via the Trainer API on four biosample-specific datasets: HeLa (BioS2), neural progenitor cells (NPC, BioS45), HepG2 (BioS73), and K562 (BioS74). Sequences were cleaned to remove ambiguous bases (Ns) and split into training, validation, and test sets. Model-specific tokenizers processed sequences with a maximum input length of 512 tokens. For non-fine-tuned models like SEI, ChromBPNet, and Enformer, the same 1 kbp sequence length was used with padding when appropriate.

Key hyperparameters included a batch size of 8, a learning rate of 1 × 10^−5^, and a maximum of 200 epochs. Early stopping with a patience of 5 epochs was used to avoid overfitting, with progress logged every 500 steps. Fine-tuned models were saved in output directories named by model checkpoint and biosample ID and uploaded to the Hugging Face Hub for reproducibility. GPU memory was cleared after each session for efficiency. This workflow enabled the adaptation of general-purpose DNA language models for cell-type-specific regulatory prediction while ensuring consistency with non-fine-tuned models.

Training and fine-tuning were performed on the NIH BioWulf high-performance computing clusters equipped with A100 GPUs, which enabled efficient processing of large-scale datasets (e.g., ~130,000 sequences of positive and control enhancer regions). Full training runs typically completed within a few hours using 8 A100 GPUs, while fine-tuning on cell line–specific subsets required proportionally less time and computational resources. CNN-based models (SEI, ChromBPNet): These models were the most computationally efficient, with training converging within 2–4 h on ~130 k sequences. Fine-tuning on smaller, cell line–specific subsets (10–30 k sequences) often required less than 2 h on a single A100 GPU, highlighting their suitability for rapid retraining and deployment. Hybrid models (Borzoi, HyenaDNA, Caduceus): Hybrid architectures combining convolutional and attention/state-space components required moderately more resources than CNNs but remained efficient. Full training typically required 4–6 h with 8 GPUs, while fine-tuning was feasible within 1–2 h using 2–4 GPUs. Transformer-based models (Nucleotide Transformer, DNABERT-2, GENA-LM, Geneformer) required substantially greater computational resources due to their parameter size and sequence length handling. Fine-tuning the larger variants (e.g., Nucleotide Transformer multi-species, GENA-LM large) could take 6–8 h across 4 GPUs. Long-range attention models, Enformer and Borzoi, designed to model >100 kb genomic contexts, were the most computationally demanding, 4 GPUs and ~8–10 h for large datasets.

### 4.3. Validation and Post-Processing

During the fine-tuning process, the models were optimized to distinguish enhancer sequences from control sequences. In contrast, the validation process focused on evaluating the difference between the reference enhancer sequence and the alternative sequence, where the middle nucleotide was mutated. The difference in output scores between these two sequences, obtained from the fine-tuned model, allowed for the estimation of the variant’s effect on the enhancer. If the output scores for both sequences were identical, the variant had no effect. Conversely, a change in output scores indicated either up- or down-regulation.

The prediction process involved generating regulatory effect predictions for each dataset by calculating the log2 ratio of alternative to reference allele probabilities for each variant. This metric provided a standardized approach to assess the relative regulatory effects of alternative alleles compared to reference alleles. Since each fine-tuned model produced logits for each class (enhancer or not), predictions for both reference and alternative alleles were processed by first calculating margin logits, which represent the difference in predicted class probabilities. These logits were then transformed into probabilities using the sigmoid function. The log2 ratio of these probabilities was computed to quantify the regulatory differences between the two alleles. Additionally, precomputed predictions stored in pickle files were loaded for specific models and datasets. The predictions for reference and alternative alleles were used to compute a ratio, representing the alternative-to-reference prediction ratio. This ratio was subsequently log2-transformed to ensure consistency in the output format. Additional preprocessing steps were applied to specific datasets as needed, such as loading specialized prediction files.

The prediction pipeline was applied across all datasets by iterating through experiments defined in a metadata table. For each experiment, data were preprocessed, and predictions were either computed or loaded accordingly. The results were compiled into a unified dictionary containing log2 prediction ratios for all variants across experiments. As in the fine-tuning process, the validation of variant/reference sequences was performed using 1 kb sequences, ensuring consistency in genomic context and input length across models. Several metrics were employed to evaluate model performance, including the Area Under the Curve (AUC), Precision-Recall Curve (PRC), F1-score, and correlation coefficients such as Pearson and Spearman. These metrics provided a comprehensive assessment of model performance, encompassing both classification accuracy and the correlation between predicted and observed values.

### 4.4. Deep Learning Models

This study utilizes several deep learning models designed for genomic sequence analysis, with a particular focus on applications to the Homo sapiens genome. The models represent the forefront of innovation in deep learning for genomics, leveraging diverse architectures and pre-training paradigms to handle the complexity of human genomic data (Table 3). To maintain focus on models explicitly designed for the human genome, this study excluded models like EVO and GPN, which are primarily aimed at other species or biological domains. 

### 4.5. Training Data

We constructed a dataset of positive and control enhancer sequences from four human cell lines: HepG2, Hela, K562, and Neural Progenitor Cells (NPCs). These cell lines were selected for their relevance in genomic research and the availability of comprehensive epigenomic data, such as DNase-Seq and histone modification profiles, sourced from the ENCODE project using genome annotations for hg19 and hg38.

Positive enhancer sequences were defined through a two-step process. First, open chromatin regions were identified using DNase-Seq data. These regions were then intersected with H3K27ac histone modification signals, a hallmark of active enhancers. To refine the dataset, we excluded non-enhancer regions such as exonic regions (coding sequences), promoter regions (near transcription start sites), and low-confidence regions listed in the ENCODE Blacklist (e.g., repetitive sequences). Specific blacklisted peaks from UCSC browser tables were also removed.

Control sequences were selected using a similar filtering process but specifically excluded regions overlapping both DNase-Seq peaks and H3K27ac signals to ensure they represented non-active genomic regions. Both positive and control sequences underwent identical exclusion criteria to eliminate confounding genomic features such as exons and promoters.

All sequences were standardized to 1 kb in length to balance sufficient coverage of regulatory regions with computational efficiency. This size is widely used in enhancer studies as it captures relevant regulatory elements while avoiding extraneous genomic features. The dataset includes an equal number of positive and control sequences for each cell line: HepG2 (14,062 each), NPC (12,466 each), K562 (19,968 each), and Hela (31,247 each). Metadata from the ENCODE project was used to retrieve biosample IDs and associated DNase-Seq and histone modification data. Reference files provided coordinates for exons, blacklist regions, and promoters, ensuring only valid enhancer or control regions were included in the final dataset.

### 4.6. Validation Data

To systematically characterize the functional effects of genetic variants on gene regulation, we compiled a diverse set of high-throughput reporter assay datasets spanning multiple cell types, genomic contexts, and experimental designs (Table 4).

Datasets 1 and 2 were sourced from the study by [35], which utilized the Survey of Regulatory Elements (SuRE) reporter assay to identify reporter assay quantitative trait loci (raQTLs)—SNPs influencing regulatory element activity. For K562 cells, 19,237 raQTLs were identified with an average allelic fold-change of 4.0-fold, while HepG2 cells yielded 14,183 raQTLs with a higher average fold-change of 7.8-fold. Most raQTLs were cell-type-specific, showing limited overlap between the two cell lines.

Dataset 3 was derived from [36] and focuses on a ~600 bp enhancer region at the SORT1 locus (1p13). This region includes SNP rs12740374, which creates a C/EBP binding site that influences SORT1 expression. The enhancer was tested in HepG2 cells using a luciferase reporter assay. Data for flipped sequences were excluded due to the orientation-independent nature of enhancers, and deletion datasets were omitted due to computational model constraints, which focus on single-nucleotide variants.

Dataset 4, from [37] used MPRA to investigate 2756 variants linked to red blood cell traits in K562 cells. The study identified 84 highly significant SNPs with functional effects, enriched in open chromatin regions shared between K562 cells and primary human erythroid progenitors (HEPs). This dataset provides insights into erythroid-specific regulatory activity.

Dataset 5, generated by [38], includes MPRA results for 284 SNPs tested in HepG2 cells. These SNPs, located within regions of high linkage disequilibrium identified through eQTL analyses, offer a detailed map of allele-specific regulatory effects relevant to liver-specific gene regulation and genotype-phenotype associations.

Dataset 6, from [39], includes lentiMPRA results for 14,042 single-nucleotide variants fixed or nearly fixed in modern humans but absent in archaic humans (e.g., Neanderthals). Tested across three cell types—embryonic stem cells, neural progenitor cells, and fetal osteoblasts—the study identified 1791 sequences with regulatory activity, including 407 with differential expression between modern and archaic humans. This dataset focuses on NPC data.

Datasets 7, 8, and 9, from [40], include MPRA results for three coding exons—SORL1 exon 17, TRAF3IP2 exon 2, and PPARG exon 6—tested in HeLa cells. Respectively, these datasets cover 1962, 1614, and 1665 SNPs. The study explored enhancer activity within these coding exons (eExons), finding that ~12% of mutations altered enhancer activity by at least 1.2-fold, with ~1% causing changes of less than 2-fold.

## Figures and Tables

**Table 1:** Pearson correlation coefficient for various deep learning models across four cell lines: K562 (19,321 SNPs), HepG2 (16,255 SNPs), NPC (14,042 SNPs), and HeLa (5241 SNPs). Standard Error is reported in brackets. Bold and underline styles denote the top and second-highest correlations per cell line, respectively.

	Cell Lines			
Models	K562 (19,321 SNPs)	HepG2 (16,255 SNPs)	NPC (14,042 SNPs)	Hela (5241 SNPs)
DNABERT-2	0.086 (0.039)	0.096 (0.191)	−0.004 (0.007)	0.120 (0.140)
NT v2-50m-ms	0.147 (0.058)	0.103 (0.036)	0.020 (0.003)	0.074 (0.047)
NT v2-100m-ms	0.152 (0.065)	0.127 (0.050)	0.016 (0.003)	0.042 (0.027)
NT v2-250m-ms	0.166 (0.064)	0.110 (0.020)	0.023 (0.003)	0.030 (0.018)
NT v2 500m-ms	0.199 (0.036)	0.114 (0.007)	0.028 (0.003)	0.036 (0.011)
NT 500m1000g	0.123 (0.084)	0.116 (0.023)	0.001 (0.002)	0.022 (0.018)
NT 500m-h-ref	0.149 (0.139)	0.119 (0.038)	0.013 (0.001)	0.067 (0.043)
NT 2.5b-1000g	0.147 (0.052)	0.112 (0.021)	0.005 (0.003)	0.087 (0.105)
NT 2.5b-m-s	0.153 (0.055)	0.139 (0.027)	0.027 (0.001)	0.066 (0.027)
Geneformer	0.005 (0.198)	0.027 (0.160)	−0.002 (0.138)	−0.007 (0.057)
Gena LM-base	0.077 (0.042)	0.050 (0.033)	0.024 (0.002)	0.029 (0.227)
Gena LM large	0.117 (0.044)	0.088 (0.036)	0.030 (0.002)	0.069 (0.216)
Gena LM b-multi	0.077 (0.051)	0.062 (0.024)	0.010 (0.001)	0.026 (0.061)
Gena LM bigbird	0.136 (0.055)	0.099 (0.029)	0.021 (0.002)	0.045 (0.015)
Hyenadna 32 k	0.084 (0.071)	0.098 (0.061)	0.002 (0.002)	0.031 (0.013)
Hyenadna 160 k	0.148 (0.053)	0.142 (0.040)	−0.007 (0.003)	0.046 (0.018)
Hyenadna 450 k	0.077 (0.074)	0.134 (0.050)	−0.007 (0.003)	0.055 (0.015)
Hyenadna 1 mf	0.118 (0.056)	0.136 (0.059)	0.001 (0.003)	0.046 (0.013)
Caduceus	0.133 (0.030)	0.108 (0.021)	0.023 (0.004)	0.020 (0.022)
ChromBPNet	0.287 (0.020)	0.295 (0.021)	0.080 (0.001)	0.289 (0.023)
TREDNet	0.315 (0.025)	0.314 (0.018)	0.075 (0.003)	0.076 (0.063)
SEI	0.297 (0.022)	0.298 (0.029)	0.073 (0.003)	0.280 (0.008)
Enformer	0.059 (0.059)	0.141 (0.110)	0.008 (0.080)	0.245 (0.027)
Borzoi	0.033 (0.016)	0.125 (0.025)	0.023 (0.004)	−0.066 (0.005)

**Table 2:** Adaptation strategies for each deep learning model used in enhancer classification and variant effect prediction tasks. The table outlines how each model was adapted or applied to handle 1 kb genomic sequences, including preprocessing steps, tokenizer usage, fine-tuning status, and inference approaches. References correspond to the original sources describing each model.

Models	Adaptation Strategy
DNABERT-2 [20,21]	Directly fine-tuned using Hugging Face’s ‘Trainer’ on 1 kb genomic sequences using the pre-trained BPE tokenizer. Model input length was restricted to 512 tokens. Adapted for binary classification (enhancer vs. control) across biosamples. Its ALiBi positional encoding and Flash Attention enabled efficient fine-tuning without modification to input format.
Nucleotide Transformer [22,23]	Adapted using Hugging Face’s ‘Trainer’ with 6-mer tokenization on 1 kb enhancer/control sequences. The tokenizer processed sequences into 512 tokens. No architectural changes were required, and the model’s multi-species pretraining proved robust for human sequence classification.
Geneformer [26]	Originally trained on single-cell transcriptomic data using gene-level inputs, Geneformer was adapted for genomic language modeling by tokenizing 1 kb enhancer/control sequences using the pretrained vocabulary and tokenizer. Tokens mapped sequence chunks to “gene-like” units, enabling use of the Transformer for DNA sequence classification. Fine-tuning was performed using Hugging Face’s ‘Trainer’, with input sequences truncated or padded to 512 tokens. Despite domain shift, the model generalized well.
GENA-LM [32]	Fine-tuned on 1 kb genomic sequences using its native sparse attention tokenizer (BigBird). Used Hugging Face-compatible tokenization pipelines for BERT-base, BERT-large, and BigBird variants. Hugging Face ‘Trainer’ enabled flexible training for binary classification. Input sequences were processed to fit within the model’s max length.
Enformer [25]	As the model was designed for very long sequences (up to 200 kb), adaptation involved truncating input to 1 kb and padding as needed. Preprocessing followed the authors’ GitHub pipeline with minimal changes [25]. No fine-tuning was performed—predictions were extracted directly from the pre-trained model for enhancer/control sequences.
HyenaDNA [33]	Used pre-trained weights and inference pipeline from the official GitHub repository. Inputs were standardized to 1 kb and padded to reach 32,768 tokens, as required by the model’s architecture. Model was used in inference-only mode to evaluate predictions without fine-tuning.
Borzoi [34]	Applied directly on 1 kb input sequences using the GitHub-published inference code. Adaptation involved formatting enhancer/control sequences to match input requirements (one-hot or token-based). The model was not fine-tuned but used as-is for classification via regression output interpretation.
Caduceus [24]	Adopted the PS version of Caduceus from the Hugging Face platform and appended a classification layer to predict the probability that a 1kb input sequence functions as an enhancer.
ChromBPNet [19]	Employed the official ChromBPNet model available on GitHub and utilized its evaluation API to compute variant effect scores based on the 1kb input sequence.
SEI [17]	Used as a non-fine-tuned model. 1 kb input sequences were one-hot encoded and passed through the SEI inference pipeline, which includes dilated convolutional layers for multi-scale pattern detection. Outputs were extracted from the final regulatory activity scores across 40+ cell types.
TREDNet [18]	Adapted for enhancer classification and SNP prioritization using the published pipeline. The model was inference-only; sequences were formatted to one-hot encoding and input into the TREDNet CNN. Saturated mutagenesis was used to evaluate variant effects post-classification.

**Table 3:** Models, architectures, training details, and applications of the models adopted.

Model	Architecture	Training Details	Applications
DNABERT-2	Transformer-based with ALiBi and Flash Attention	Pre-trained using Byte Pair Encoding (BPE) tokenization; compact representation of sequences; optimized for computational efficiency and scalability.	Transformer-based model pre-trained on k-mers; well suited for sequence classification, motif discovery, and variant effect prediction across species, with strong performance on short-range motif detection.
Nucleotide Transformer (8 models, 2 versions)	Transformer-based (50 M–2.5 B parameters)	Pretrained on multi-species genomes with 6-mer tokenization; trained on 300B tokens using Adam optimizer with warmup and decay schedules; supports long-range genomic dependencies.	Large-scale Transformer trained on human and multi-species genomes; supports comparative genomics, functional annotation, and variant effect prediction; versions range from smaller models for standard GPUs to billion-parameter models for large-scale training, and species-specific vs. multi-species training.
Geneformer	Transformer-based with rank-based analysis	Pretrained on single-cell transcriptome datasets using masked gene prediction; optimized for noise resilience and stability in single-cell data.	Transformer optimized for single-cell data; captures gene network dynamics and enables single-cell state classification; available in versions pre-trained on different single-cell datasets.
GENA-LM (4 models)	BERT-base, BERT-large, BigBird variants	Pretrained on genomic sequences using sparse attention mechanisms (BigBird); supports long-range sequence modeling and local feature detection; memory augmentation techniques applied for scalability.	Transformer optimized for single-cell data; captures gene network dynamics and enables single-cell state classification; available in versions pre-trained on different single-cell datasets.
Enformer	Convolution + Transformer hybrid	Pretrained on DNA sequences up to 200,000 bp using convolutional blocks for spatial reduction and Transformer layers for long-range interactions; predicts over 5000 epigenetic features.	Attention-based architecture capable of modeling >100 kb genomic context; excels at gene expression prediction and regulatory element analysis; distributed in full and lightweight implementations.
HyenaDNA (4 models)	Runtime-scalable models	Pretrained on human reference genome with extended sequence lengths (32,000–1,000,000 bp); optimized for runtime scalability using AWS HealthOmics and SageMaker infrastructure.	Hybrid convolutional/state-space model for long-range genomic interaction analysis; computationally efficient for long sequences, with model size scalable to hardware constraints.
Caduceus	Transformer-based model with position-aware embeddings and rotary attention	Pretrained on large-scale human genomic sequences in a self-supervised manner using masked language modeling.	General-purpose foundation model for regulatory genomics; trained on large-scale datasets; offers different checkpoints for diverse regulatory genomics tasks.
ChromBPNet	CNN with dilated convolutions and residual connections	Trained on chromatin accessibility data (e.g., ATAC-seq or DNase-seq).	CNN-based model designed for predicting chromatin accessibility; base-resolution DNase/ATAC signal prediction; relatively lightweight and accessible for most hardware setups.
Borzoi	Multi-layer regulatory model	Trained to predict RNA-seq coverage directly from DNA sequences; integrates multiple layers of regulatory predictions; fine-tuned for tissue-specific applications.	Hybrid CNN/attention model for cell- and tissue-specific RNA-seq coverage prediction and cis-regulatory pattern detection; released in different model sizes to balance accuracy with resource use.
SEI	Residual dilated convolutional layers	Trained on one-hot encoded sequences (4096 bp) using residual paths for multi-scale pattern detection; incorporates global integration layers for long-range dependencies; balances computational efficiency with expressiveness.	CNN-based sequence-to-function model for variant effect prediction on cis-regulatory activity across chromatin profiles; robust across cell types without fine-tuning, available in multiple pre-trained versions.

**Table 4:** Overview of the datasets used to assess regulatory variant function across diverse cell types and genomic contexts.

Dataset [ref]	Cell Type	Assay Type	No. of Variants/SNPs	Key Findings
Dataset 1 [35]	K562	SuRE reporter assay	19,237 raQTLs	Mean allelic fold-change: 4.0-fold. Most raQTLs are cell-type-specific.
Dataset 2 [35]	HepG2	SuRE reporter assay	14,183 raQTLs	Mean allelic fold-change: 7.8-fold. Limited overlap with K562 raQTLs, indicating cell-type specificity.
Dataset 3 [36]	HepG2	Luciferase reporter	1 enhancer (SORT1, ~600 bp, incl. rs12740374)	SNP rs12740374 creates C/EBP binding site affecting SORT1 expression. Flipped and deletion data excluded.
Dataset 4 [37]	K562	MPRA	2756 variants	84 significant SNPs with functional effects, enriched in open chromatin shared with human erythroid progenitors (HEPs).
Dataset 5 [38]	HepG2	MPRA	284 SNPs	SNPs in high LD regions from eQTL analyses. Provides allele-specific regulatory effects relevant to liver gene regulation.
Dataset 6 [39]	NPC	lentiMPRA	14,042 SNVs	Variants fixed/nearly fixed in modern humans (absent in archaic). 1791 with regulatory activity; 407 show differential expression. Focus on NPC data.
Dataset 7 [40]	HeLa	MPRA coding exon	1962 SNPs (SORL1 exon 17)	~12% of mutations alter enhancer activity by ≥1.2-fold; ~1% by <2-fold.
Dataset 8 [40]	HeLa	MPRA coding exon	1614 SNPs (TRAF3IP2 exon 2)	Same as above—functional testing of coding exon enhancer activity.
Dataset 9 [40]	HeLa	MPRA coding exon	1665 SNPs (PPARG exon 6)	Same as above—functional testing of coding exon enhancer activity.

## Data Availability

Code, data, and analysis repository: https://github.com/tanoManzo/AI4Genomic.git. Fine-tuned models: https://huggingface.co/tanoManzo. (Access data: 5 September 2025).

## Supplementary Materials

The following supporting information can be downloaded at: https://www.mdpi.com/article/10.3390/genes16101223/s1, Figure S1: Spearman correlation between model predictions and experimental log2-fold changes for enhancer variant effects across the human genome. Bar colors denote model architectures (CNN: green, transformer: blue, and hybrid: orange). All correlations have *p*-values < 0.05, and error bars show variance; Figure S2: Heatmap of model variant predictions (Predicted) versus experimental values (Actual Results) at different p-value thresholds, highlighting performance variations in identifying positive/negative outcomes across architectures.. The color intensity represents the fraction of values predicted as positive/negative relative to the experimental positive/negative values. Red indicates higher fractions (desired in the first two columns from the left), while blue indicates lower fractions (desired in the last two columns); Table S1: Spearman correlation for various deep learning models across four cell lines: K562 (19,321 SNPs), HepG2 (16,255 SNPs), NPC (14,042 SNPs), and HeLa (5241 SNPs). Bold and underline styles denote the top and second-highest correlations per cell line, respectively; Table S2: Pearson correlation coefficients for various deep learning models across multiple datasets (Datasets 1–9). Bold and underline denote the top and second-highest correlations per cell line; Table S3: Spearman correlation for various deep learning models across multiple datasets (Data 1–9 and the respective SNPs). Bold and underline styles denote the top and second-highest correlations per cell line, respectively; Figure S3a–d: Model performance relative to experimental data significance, using top models from each architecture category; Figure S4: Comparison of model-predicted versus experimentally observed log2 fold-change values for ~14,000 SNPs from Dataset 2 (HepG2 cell line). Each panel shows a scatterplot comparing the predicted (x-axis) and experimental (y-axis) log2 fold changes. Models shown represent the top-performing model from each architectural category: TREDNet (CNN), Hyenadna (Hybrid), and Nucleotide Transformer v2 (Transformer). The top row includes all data except experimental values with low variance around zero (filtered with ε = 1). The middle row retains experimental values but removes predicted values near zero. The bottom row removes both predicted and experimental low-variance values. Diagonal lines indicate ideal agreement, and points are colored by variant direction, allowing visual comparison of prediction quality across model types under increasingly stringent filtering; Figure S5: Impact of Input Sequence Length on Model Correlation Performance. Comparison of Pearson and Spearman correlation coefficients (with corresponding p-values annotated) across different input sequence lengths (from 1000 bp to 6000 bp) for the best-performing Transformer model, Nucleotide Transformer v2; Figure S6: Performance of different deep learning architectures on variant effect prediction. The models were trained on data from different cell lines than the evaluation dataset, simulating a zero-shot setting particularly relevant for Transformer-based models.
