# Supplementary material for: Comparative Analysis of Deep Learning Models for Predicting Causative Regulatory Variants
Source: Genes (Basel). 2025 Oct 15;16(10):1223. doi: 10.3390/genes16101223 (PMC12562713; doi:10.3390/genes16101223)
Supplement: Supplementary file 1 [file genes-16-01223-s001.zip › genes-3887697-supplementary.pdf]

Comparative Analysis of Deep Learning Models for Predicting Causative Regulatory Variants – Supplementary Materials

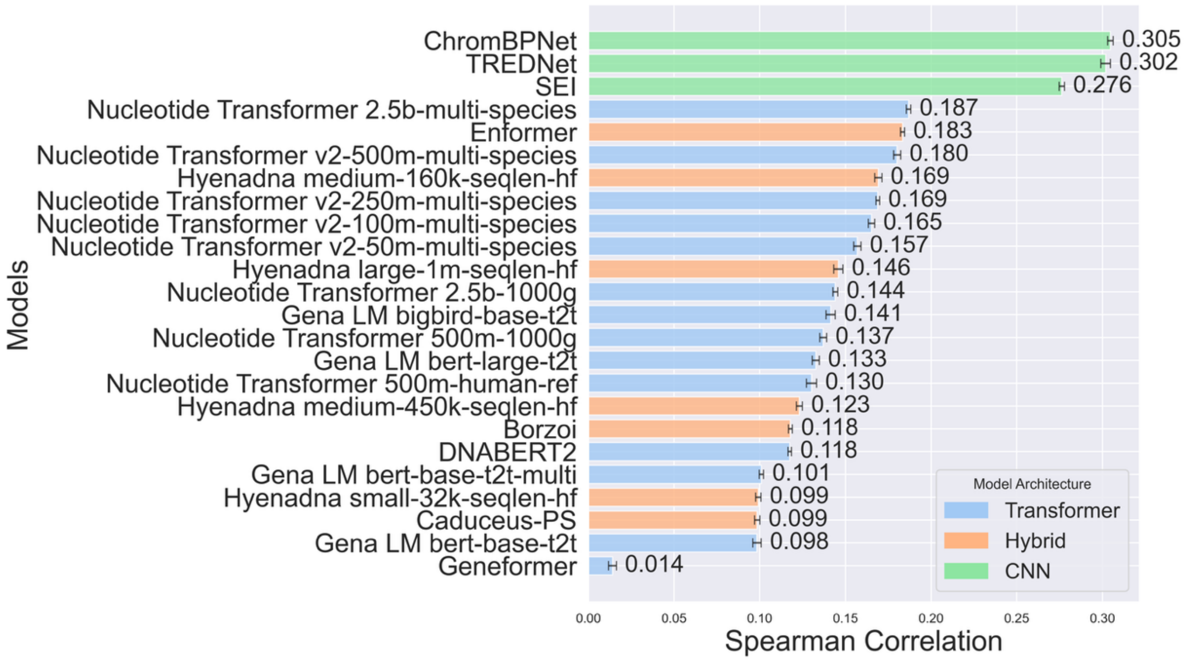

Figure S1: *Spearman correlation between model predictions and experimental log2-fold changes for enhancer variant effects across the human genome. Bar colors denote model architectures (CNN: green, transformer: blue, and hybrid: orange). All correlations have p-values < 0.05, and error bars show variance.*

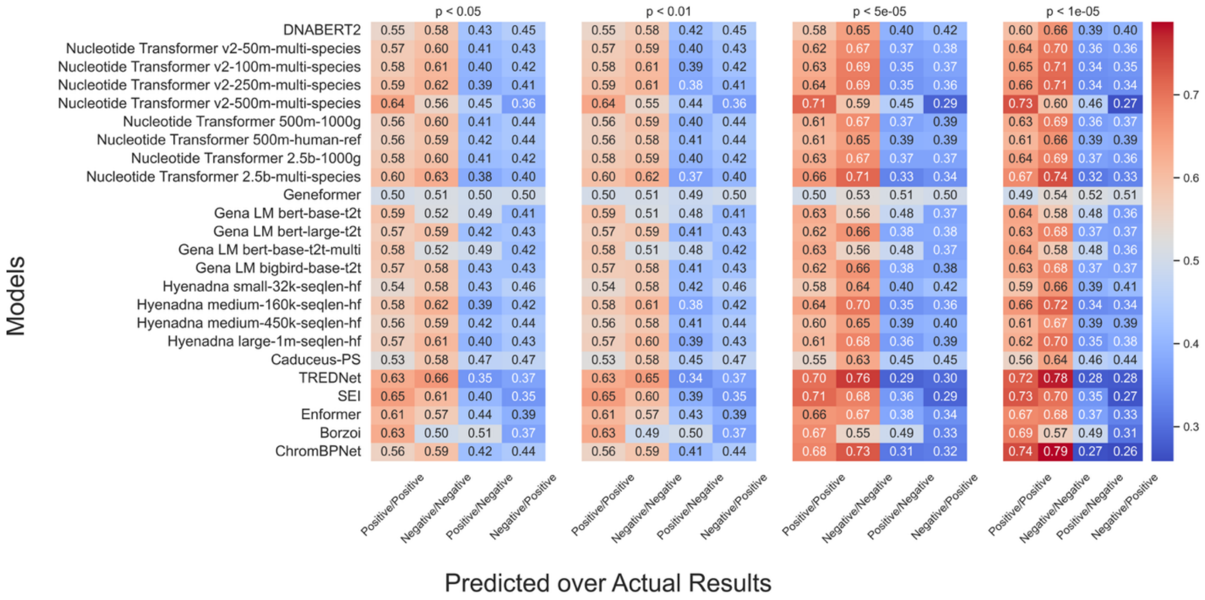

Figure S2: *Heatmap of model variant predictions (Predicted) versus experimental values (Actual Results) at different p-value thresholds, highlighting performance variations in identifying positive/negative outcomes across architectures.. The color intensity represents the fraction of values predicted as positive/negative relative to the experimental positive/negative values. Red indicates higher fractions (desired in the first two columns from the left), while blue indicates lower fractions (desired in the last two columns).*

Table S1: *Spearman correlation for various deep learning models across four cell lines: K562 (19321 SNPs), HepG2 (16255 SNPs), NPC (14042 SNPs), and HeLa (5241 SNPs). Bold and underline styles denote the top and second-highest correlations per cell line, respectively.*

	Cell Lines			
**Models**	**k562 (19321 SNPs)**	**hepg2 (16255 SNPs)**	**NPC (14042 SNPs)**	**Hela (5241 SNPs)**
DNABERT2	0.136255	0.13844	0.00983	0.10131
Nucleotide Transformer v2-50m-multi-species	0.193606	0.176066	0.029745	0.091993
Nucleotide Transformer v2-100m-multi-species	0.194085	0.210449	0.029202	0.085566
Nucleotide Transformer v2-250m-multi-species	0.230211	0.178415	0.031789	0.091625
Nucleotide Transformer v2-500m-multi-species	0.255502	0.193536	0.039783	0.076763
Nucleotide Transformer 500m-1000g	0.152783	0.193222	0.010707	0.06466
Nucleotide Transformer 500m-human-ref	0.158163	0.17474	0.016139	0.058858
Nucleotide Transformer 2.5b-1000g	0.167759	0.181192	0.014801	0.081246
Nucleotide Transformer 2.5b-multi-species	0.226941	0.22058	0.016179	0.091921
Geneformer	0.00589	0.030644	-0.00983	-0.009303
Gena LM bert-base-t2t	0.122552	0.116882	0.025676	0.052523
Gena LM bert-large-t2t	0.169897	0.142243	0.041452	0.084151
Gena LM bert-base-t2t-multi	0.096464	0.140658	0.015713	0.047429
Gena LM bigbird-base-t2t	0.169682	0.159723	0.023648	0.088436
Hyenadna small-32k-seqlen-hf	0.110639	0.129993	0.000771	0.075399
Hyenadna medium-160k-seqlen-hf	0.1927	0.208351	0.007788	0.118122
Hyenadna medium-450k-seqlen-hf	0.115686	0.186035	0.007832	0.093602
Hyenadna large-1m-seqlen-hf	0.162212	0.194539	0.012185	0.077734
Caduceus	0.153784	0.365124	0.068044	0.025176
ChromBPNet	<u>0.306716</u>	0.305535	**0.127997**	**0.189325**
TREDNet	**0.313784**	**0.365124**	<u>0.068044</u>	0.103394
SEI	0.287206	<u>0.315009</u>	0.067235	<u>0.155081</u>
Enformer	0.131957	0.230396	-0.005137	0.129005
Borzoi	0.089077	0.173757	0.024613	-0.019793

Table S2: *Pearson correlation coefficients for various deep learning models across multiple datasets (Datasets 1-9). Bold and underline denote the top and second-highest correlations per cell line.*

Model	Dataset 1	Dataset 2	Dataset 3	Dataset 4	Dataset 5	Dataset 6	Dataset 7	Dataset 8	Dataset 9
	(19237 SNPs)	(14183 SNPs)	(1789 SNPs)	(84 SNPs)	(283 SNPs)	(14042 SNPs)	(1692 SNPs)	(1614 SNPs)	(1665 SNPs)
	K562	HepG2	HepG2	G562	HepG2	NPC	Hela	Hela	Hela
DNABERT2	0.086	0.098	0.049	0.140	0.137	-0.004	0.613	0.480	0.211
Nucleotide Transformer v2-50m-multi-species	0.147	0.104	0.129	0.178	0.322	0.021	0.575	0.354	0.218
Nucleotide Transformer v2-100m-multi-species	0.152	0.128	0.113	0.207	**0.570**	0.022	0.526	0.232	0.136
Nucleotide Transformer v2-250m-multi-species	0.166	0.111	0.217	0.142	0.431	0.042	-0.383	0.306	**0.329**
Nucleotide Transformer v2-500m-multi-species	0.199	0.116	-0.030	0.389	0.028	0.084	0.261	0.264	-0.010
Nucleotide Transformer 500m-1000g	0.123	0.119	0.111	0.325	0.068	0.000	0.532	0.346	0.162
Nucleotide Transformer 500m-human-ref	0.149	0.120	0.052	0.296	0.427	0.002	0.449	0.253	0.218
Nucleotide Transformer 2.5b-1000g	0.147	0.113	0.165	0.238	0.182	0.004	0.607	0.357	0.188
Nucleotide Transformer 2.5b-multi-species	0.153	0.140	0.340	0.311	<u>0.491</u>	0.060	0.557	0.366	0.066
Geneformer	0.005	0.028	0.029	0.228	0.121	0.004	-0.108	-0.021	0.031
Gena LM bert-base-t2t	0.077	0.050	-0.008	0.151	-0.138	0.047	-0.465	0.216	0.031
Gena LM bert-large-t2t	0.117	0.089	0.192	0.203	0.234	0.041	0.754	0.099	0.017
Gena LM bert-base-t2t-multi	0.076	0.063	-0.058	0.156	-0.081	0.004	-0.400	0.057	0.107
Gena LM bigbird-base-t2t	0.136	0.100	0.291	<u>0.412</u>	-0.013	0.039	0.624	0.338	0.017
Hyenadna small-32k-seqlen-hf	0.084	0.100	0.104	0.174	0.265	0.019	0.293	0.015	0.251
Hyenadna medium-160k-seqlen-hf	0.149	0.144	0.267	0.289	0.123	-0.016	0.317	0.137	<u>0.318</u>
Hyenadna medium-450k-seqlen-hf	0.077	0.137	0.073	0.209	0.214	-0.036	0.630	0.166	0.238
Hyenadna large-1m-seqlen-hf	0.118	0.138	0.126	0.243	0.204	-0.016	0.606	0.116	0.227
Caduceus	0.133	0.010	0.036	0.118	-0.315	0.045	0.010	0.138	0.044
ChromBPNet	0.291	0.314	0.304	0.197	0.084	0.284	0.394	0.303	0.177
TREDNet	**0.316**	**0.342**	<u>0.397</u>	**0.601**	0.363	<u>0.167</u>	0.784	0.410	0.085
SEI	<u>0.298</u>	<u>0.298</u>	0.394	0.341	0.051	**0.190**	<u>0.801</u>	**0.595**	0.237
Enformer	0.058	0.137	**0.492**	0.410	0.434	0.037	**0.843**	<u>0.542</u>	0.142
Borzoi	0.033	0.125	0.012	0.306	0.121	0.050	0.231	-0.237	-0.120

Table S3: *Spearman correlation for various deep learning models across multiple datasets (Data 1-9 and the respective SNPs). Bold and underline styles denote the top and second-highest correlations per cell line, respectively.*

Model	Dataset 1 (19237 SNPs) K562	Dataset 2 (14183 SNPs) HepG2	Dataset 3 (1789 SNPs) HepG2	Dataset 4 (84 SNPs) G562	Dataset 5 (283 SNPs) HepG2	Dataset 6 (14042 SNPs) NPC	Dataset 7 (1692 SNPs) Hela	Dataset 8 (1614 SNPs) Hela	Dataset 9 (1665 SNPs) Hela
DNABERT2	0.136	0.136	0.103	0.168	0.105	0.018	0.627	0.513	0.336
Nucleotide Transformer v2-50m-multi-species	0.194	0.170	0.191	0.348	-0.018	0.029	0.273	0.496	0.252
Nucleotide Transformer v2-100m-multi-species	0.194	0.203	0.222	0.135	<u>0.382</u>	0.016	0.545	0.442	0.069
Nucleotide Transformer v2-250m-multi-species	0.231	0.171	0.306	0.196	0.372	0.027	0.309	0.507	0.186
Nucleotide Transformer v2-500m-multi-species	0.256	0.187	-0.020	0.442	0.226	0.079	0.264	0.432	-0.089
Nucleotide Transformer 500m-1000g	0.153	0.188	0.148	0.429	-0.077	0.014	0.345	0.390	0.064
Nucleotide Transformer 500m-human-ref	0.158	0.170	0.062	<u>0.528</u>	0.066	0.007	0.309	0.416	0.111
Nucleotide Transformer 2.5b-1000g	0.168	0.173	0.285	0.488	0.355	0.016	0.582	0.495	0.104
Nucleotide Transformer 2.5b-multi-species	0.228	0.209	0.342	0.400	0.305	0.040	0.427	0.517	0.072
Geneformer	0.006	0.030	0.044	0.330	-0.076	0.007	0.027	-0.039	-0.017
Gena LM bert-base-t2t	0.122	0.115	0.066	0.285	0.180	0.059	-0.518	0.394	0.091
Gena LM bert-large-t2t	0.170	0.135	0.221	0.510	0.360	0.047	0.555	0.448	0.199
Gena LM bert-base-t2t-multi	0.096	0.140	0.012	0.410	-0.138	0.019	-0.035	0.242	0.157
Gena LM bigbird-base-t2t	0.170	0.155	0.287	0.512	0.090	0.037	0.482	0.428	0.178
Hyenadna small-32k-seqlen-hf	0.111	0.126	0.200	0.391	0.146	0.011	0.545	-0.096	<u>0.371</u>
Hyenadna medium-160k-seqlen-hf	0.193	0.201	0.310	0.487	0.178	-0.009	0.409	0.461	**0.428**
Hyenadna medium-450k-seqlen-hf	0.116	0.181	0.147	0.274	0.167	-0.013	0.655	0.376	0.326
Hyenadna large-1m-seqlen-hf	0.162	0.188	0.182	0.453	0.270	-0.010	<u>0.700</u>	0.406	0.370
Caduceus	0.158	-0.006	0.041	0.301	-0.484	0.034	0.200	0.146	-0.092
ChromBPNet	<u>0.307</u>	0.056	0.214	0.072	0.061	**0.368**	0.263	0.111	0.162
TREDNet	**0.314**	**0.360**	0.416	**0.573**	**0.435**	0.135	0.673	0.536	0.267
SEI	0.287	<u>0.309</u>	<u>0.427</u>	0.495	0.149	<u>0.148</u>	0.627	**0.633**	0.248
Enformer	0.132	0.218	**0.566**	0.374	0.267	-0.016	**0.900**	<u>0.553</u>	0.053
Borzoi	0.088	0.172	0.036	0.270	0.173	0.073	0.282	-0.368	0.009

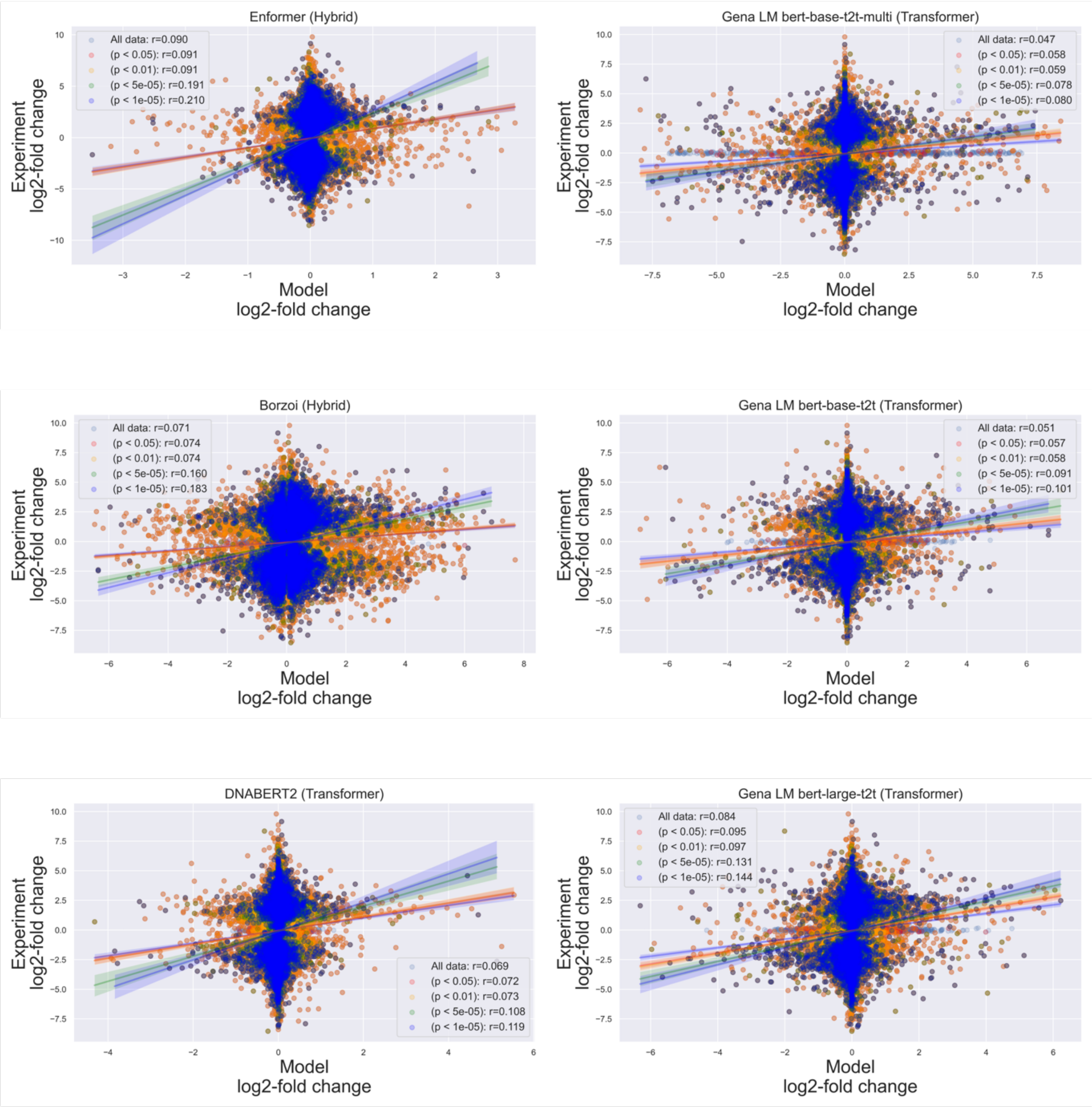

Figure S3a: *Model performance relative to experimental data significance, using top models from each architecture category.*

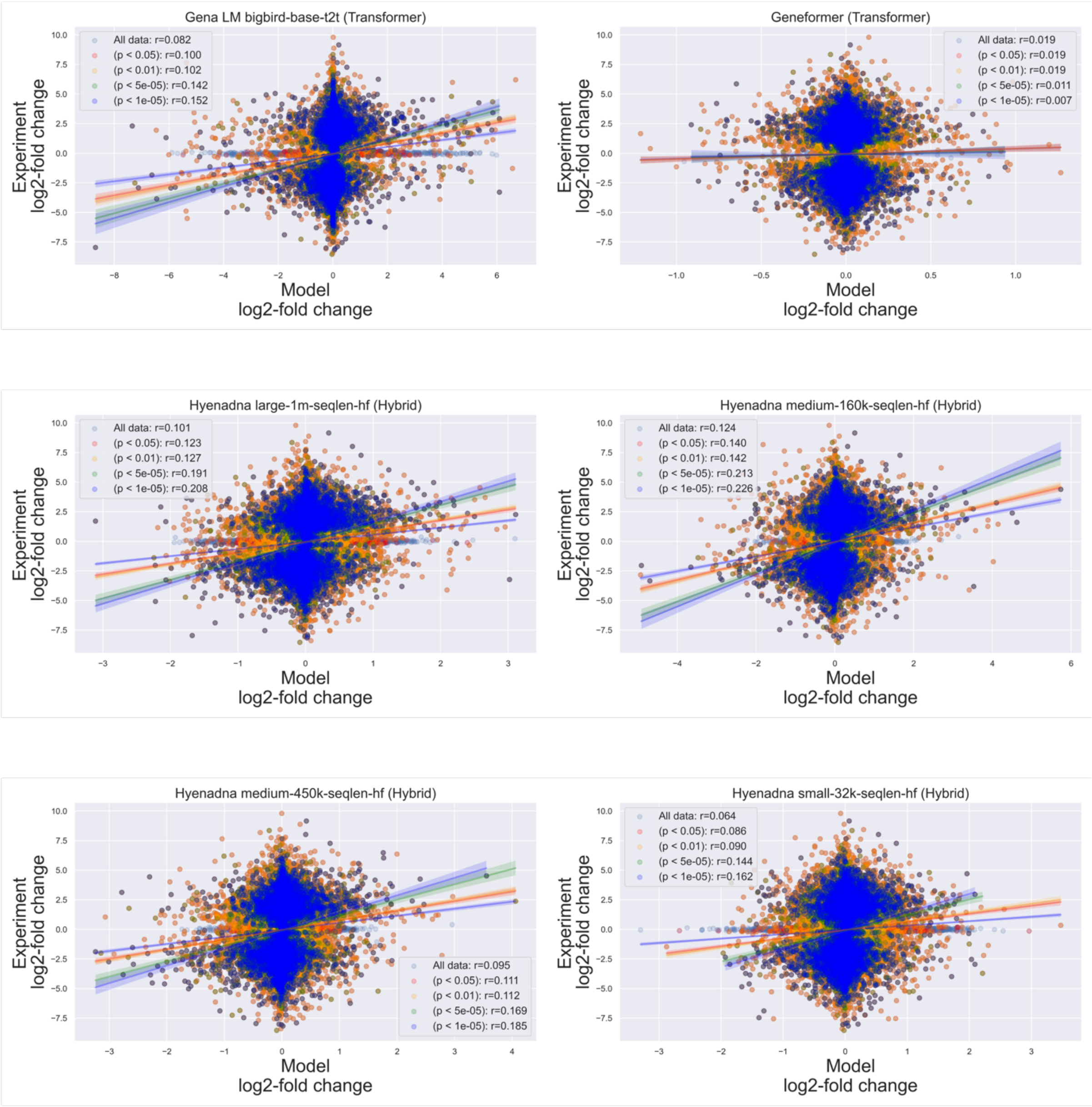

Figure S3b: *Model performance relative to experimental data significance, using top models from each architecture category.*

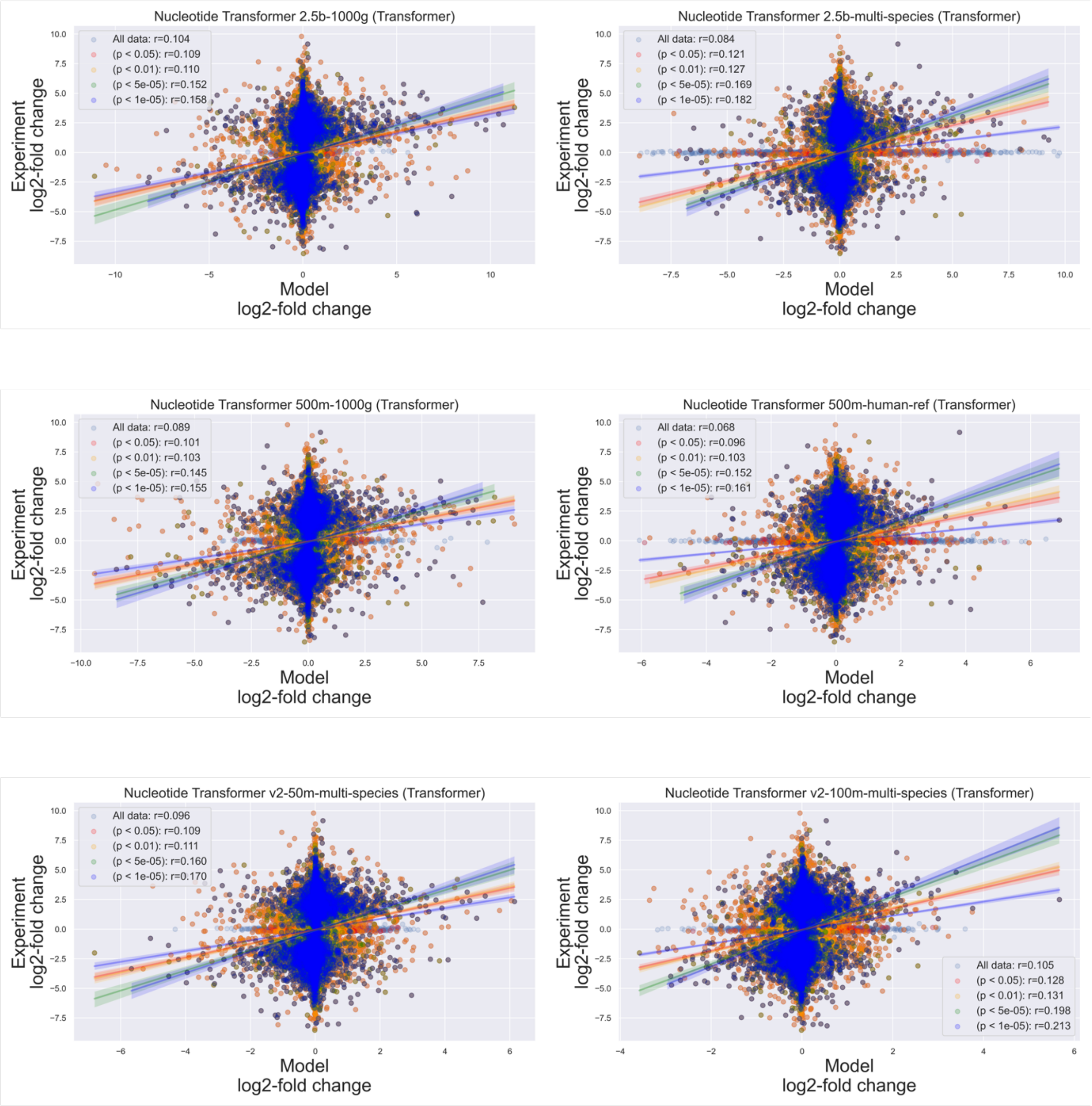

Figure S3c*: Model performance relative to experimental data significance, using top models from each architecture category.*

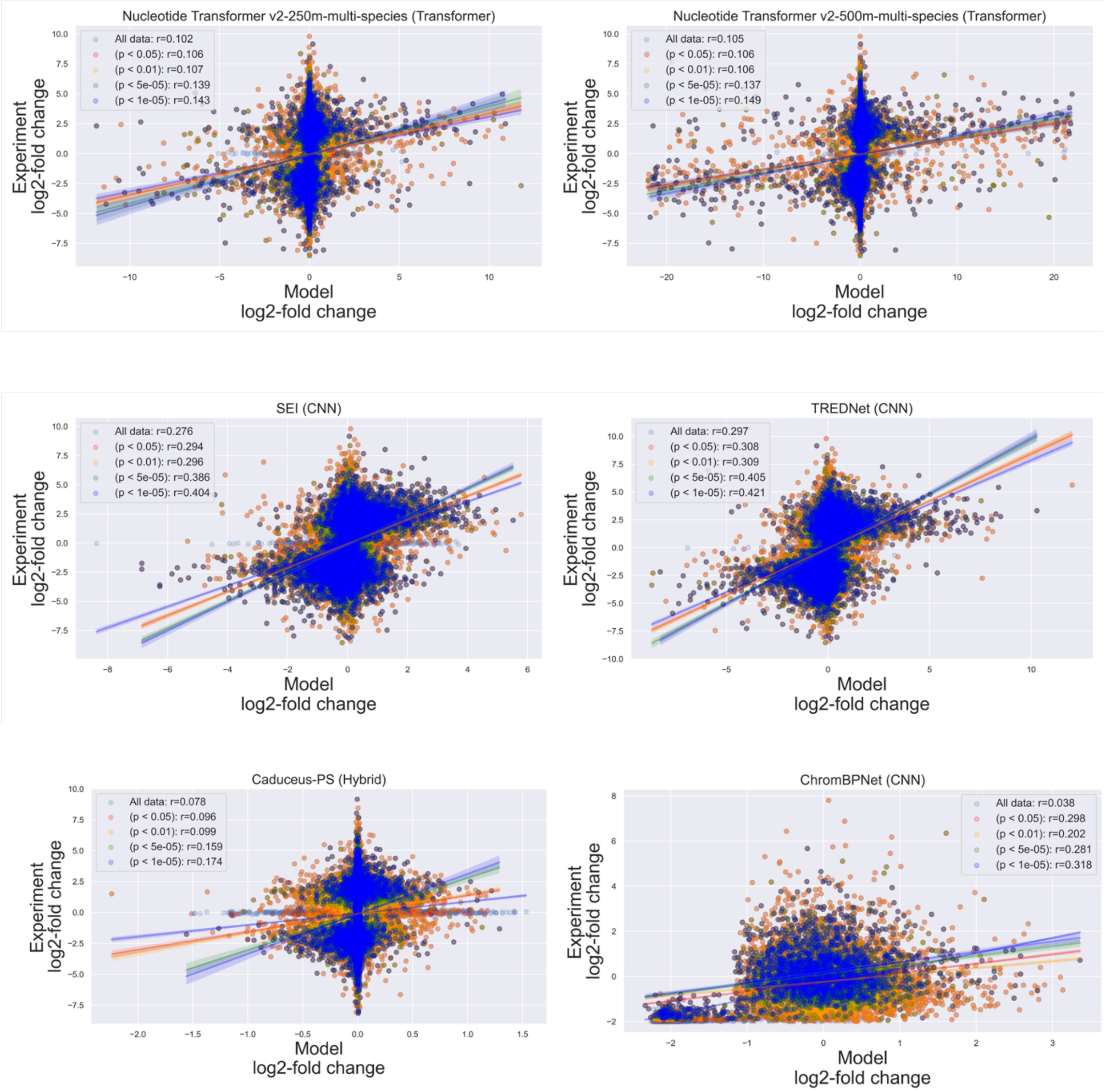

Figure S3d*: Model performance relative to experimental data significance, using top models from each architecture category.*

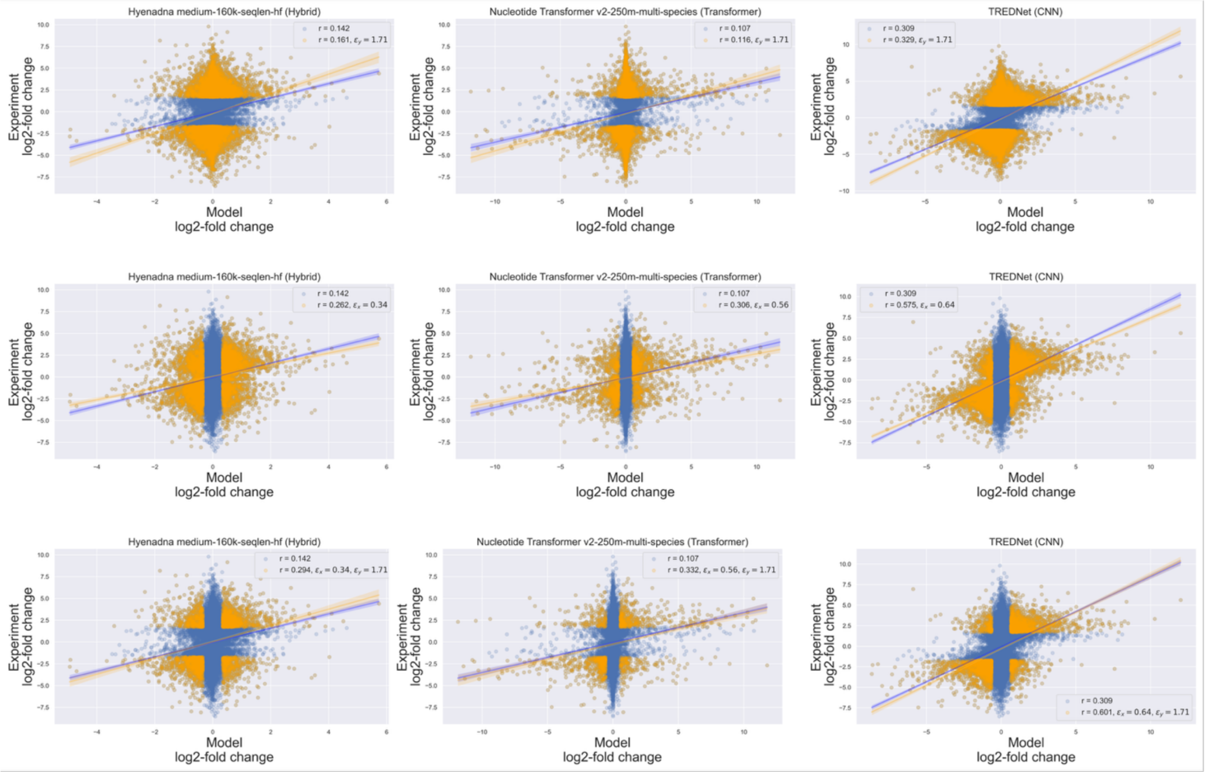

Figure S4: *Comparison of model-predicted versus experimentally observed log2 fold-change values for ~14,000 SNPs from Dataset 2 (HepG2 cell line). Each panel shows a scatterplot comparing the predicted (x-axis) and experimental (y-axis) log2 fold changes. Models shown represent the top-performing model from each architectural category: TREDNet (CNN), Hyenadna (Hybrid), and Nucleotide Transformer v2 (Transformer). The top row includes all data except experimental values with low variance around zero (filtered with ε = 1). The middle row retains experimental values but removes predicted values near zero. The bottom row removes both predicted and experimental low-variance values. Diagonal lines indicate ideal agreement, and points are colored by variant direction, allowing visual comparison of prediction quality across model types under increasingly stringent filtering.*

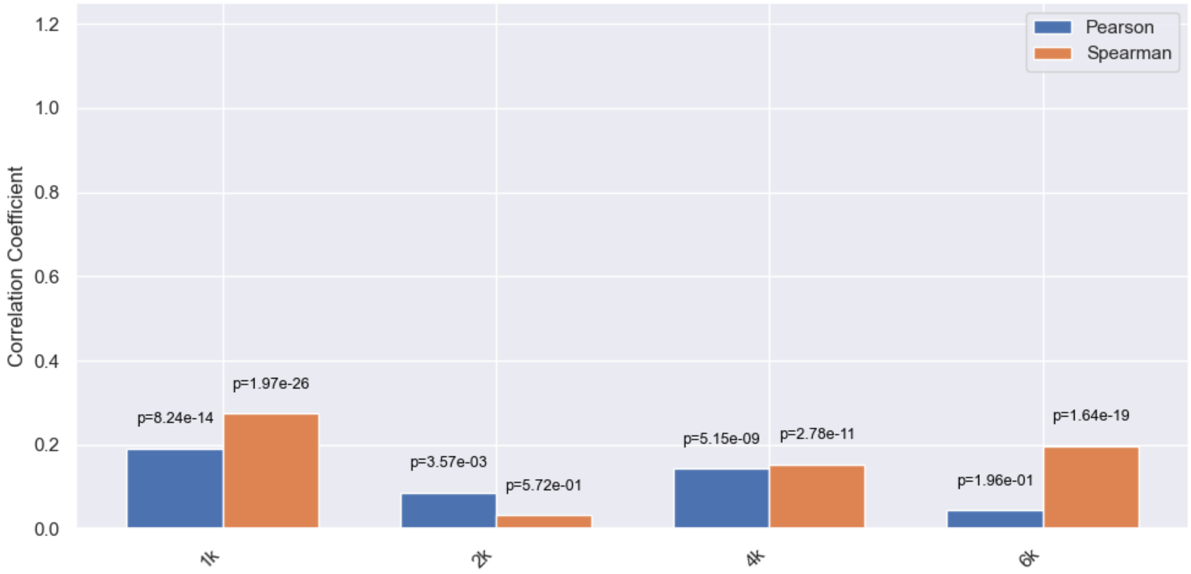

***Figure S5**: Impact of Input Sequence Length on Model Correlation Performance. Comparison of Pearson and Spearman correlation coefficients (with corresponding p-values annotated) across different input sequence lengths (from 1,000 bp to 6,000 bp) for the best-performing Transformer model, Nucleotide Transformer v2.*

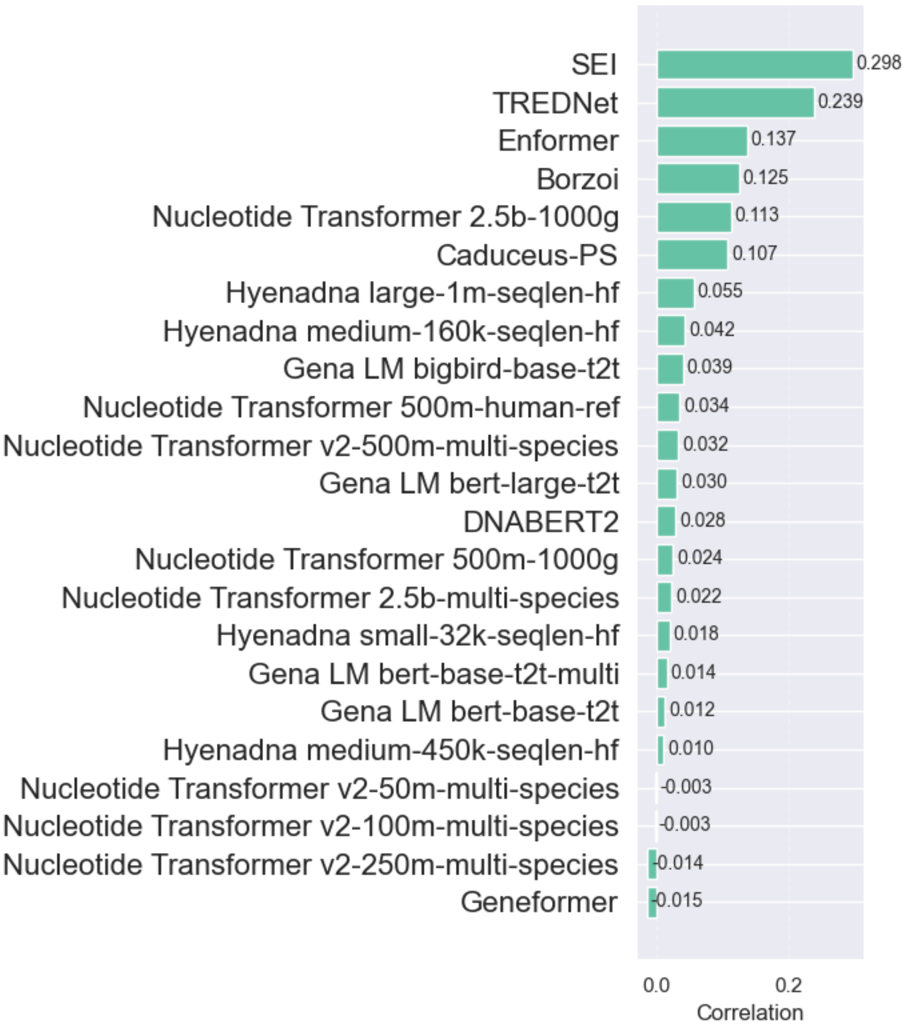

***Figure S6**: Performance of different deep learning architectures on variant effect prediction. The models were trained on data from different cell lines than the evaluation dataset, simulating a zero-shot setting particularly relevant for Transformer-based models.*
